# Improving reproducibility of data analysis and code in medical research: 5 recommendations to get started

**DOI:** 10.1136/bmjopen-2025-104691

**Published:** 2025-10-02

**Authors:** Anna Maria Streiber, Sanne J W Hoepel, Elisabet Blok, Frank J A van Rooij, Julia Neitzel, Jeremy Labrecque, M Kamram Ikram, Daniel Bos

**Affiliations:** 1 Department of Radiology and Nuclear Medicine, Erasmus MC University Medical Center Rotterdam, Rotterdam, Netherlands; 2 Department of Epidemiology, Erasmus MC University Medical Center Rotterdam, Rotterdam, Netherlands; 3 Erasmus MC University Medical Center Sophia Children's Hospital, Rotterdam, Netherlands; 4 Department of Neurology, Erasmus MC University Medical Center Rotterdam, Rotterdam, Netherlands; 5 Department of Epidemiology, Harvard T H Chan School of Public Health, Boston, Massachusetts, USA; 6 Neurosciences, KU Leuven, Leuven, Flanders, Belgium

**Keywords:** Education, Medical, EPIDEMIOLOGY, Methods, Research Design, STATISTICS & RESEARCH METHODS

## Abstract

Due to the growing use of high-dimensional data and methodological advances in medical research, reproducibility of research is increasingly dependent on the availability of reproducible code. However, code is rarely made available and too often only partly reproducible. Here, we aim to provide practical and easily implementable recommendations for medical researchers to improve the reproducibility of their code. We reviewed current coding practices in the population-based Rotterdam Study cohort. Based on this review, we formulated the following five recommendations to improve the reproducibility of code used in data analysis: (1) make reproducibility a priority and allocate time and resources; (2) implement systematic code review by peers, as it further strengthens reproducibility. We provide a code review checklist, which serves as a practical tool to facilitate structured code review; (3) write comprehensible code that is well-structured; (4) report decisions transparently, for instance by providing the annotated workflow code for data cleaning, formatting and sample selection; and (5) focus on accessibility of code and data and share both, when possible, via an open repository to foster accessibility. Ideally, this repository should be managed by the institution and should be accessible to everyone. Based on these five recommendations, medical researchers can take actionable steps to improve the reproducibility of their research. Importantly, these recommendations are thought to provide a practical starting point for enhancing reproducibility rather than mandatory guidelines.

## Introduction

 The reproducibility of research findings is a key part of the scientific method. Until recently, the reproducibility of medical research—ie, whether an independent analysis of the same data yields consistent findings^1 2^—mainly depended on the detailed description of the methods in the research paper. However, with the growing use of high-dimensional data, recent methodological advances and increasing occurrence of multi-cohort or multi-site studies, medical research has become increasingly complex. In this context, reproducibility of research strongly depends on the reproducibility of the code used in research, ie, scripts written in programming languages such as R or Python that are used to preprocess data, derive analytical datasets from larger repositories and perform statistical analyses.^1 3 4^ However, Hamilton and colleagues^5^ estimated that less than 0.5% of medical research studies that were published since 2016 shared their analytical code. Furthermore, reviews estimate that of papers that share code and data, only a fraction is reproducible, with estimates ranging widely between 17 and 82%.^6^,^8^ 

Existing recommendations for good coding practices often assume a computer science skillset (eg, Caitlin *et al* ^9 10^) and can be hard to implement for medical researchers who come from a wide range of backgrounds. To this end, we formulated five key recommendations for good coding practices for reproducible medical research. For this, we reviewed current coding practices in the literature and in the context of the population-based Rotterdam Study cohort.^11^ These recommendations do not function as guidelines that must be adhered to but rather provide medical researchers, especially early career researchers, with a range of options to get started on reproducibility. An illustrative example of several of these reproducible coding practices is available in online supplemental file 1 (code available via https://doi.org/10.5281/zenodo.16562233). While this example is written in R, a common programming language in medical research,^12^ the structure and recommendations generalise to other coding languages.

## Review of current coding practices

Reproducibility in epidemiologic research is closely tied to the quality and transparency of analytical code. However, several studies have highlighted persistent shortcomings in current practices. Petrone *et al* showed how minor differences in operational study definitions, such as cohort selection procedures, can lead to substantially different results, underscoring the need for precise and transparent reporting of coding decisions.^13^ Similarly, Laurinavichyute and colleagues emphasised that sharing well-documented code is crucial for reproducibility and identified common barriers, including outdated code, missing preprocessing scripts and insufficient documentation.^7^ A recent review of over 9000 R scripts from replication datasets found that the majority failed to run in clean environments, illustrating widespread technical and documentation issues.^14^ While these studies demonstrate that reproducibility can be improved through better coding practices, they do not always provide an in-depth evaluation of qualitative aspects of the code. To address this, we reviewed the internal code archive of the Rotterdam Study.

The Rotterdam Study is a population-based cohort study that has been ongoing since 1990.^11^ It was designed to investigate determinants and consequences of ageing and age-related disease and has a locally maintained archive of code. For each Rotterdam Study publication, a folder is stored with the original data files and scripts or syntax files necessary for performing the statistical analyses. As a team comprised of epidemiologists, quantitative researchers and a data manager working with cohort data, we examined this archive to better understand how analytical code is written and maintained in real-world epidemiologic research.

We observed two key areas for improvement, which form the basis of our recommendations. First, we found that code was often written solely for use by the author, and not with reproducibility in mind, limiting comprehensibility of the code. For example, code often lacked a clear structure, such as comments and headings. Based on our observations and on literature from the epidemiology field, we recommend researchers make reproducibility a priority (recommendation 1), implement code review (recommendation 2) and improve comprehensibility (recommendation 3). Within the Rotterdam Study, we often saw that transparency was lacking on key decisions in the analytical process. A recurring example was the lack of a detailed description of the sample selection, hindering reproducibility. Therefore, we recommend that researchers improve transparency by noting these decisions in the code (recommendation 4). Last, we realised that reproducibility of all these analyses is hampered, as code and data are only available to researchers affiliated with the Rotterdam Study. Therefore, we recommend authors and institutions to share code and data as openly as possible (recommendation 5).

## Recommendation 1: make reproducibility a priority

Making reproducibility a priority requires time and resources, which are scarce commodities among scholars. To get motivated to set reproducibility as a priority, one should appreciate the intrinsic value of reproducibility for research, as well as the key benefits it has for individual researchers. Reproducibility is an essential part of the cycle of empirical research, as it facilitates replicating findings in a new setting or extending them to test a new hypothesis. For this reason, reproducible coding practices, especially sharing data and code, are frequently recommended open science practices.^1^ Given that many research projects are publicly funded, we argue that researchers have a societal responsibility to make their methods reproducible and publicly accessible. This is also increasingly acknowledged by other parties, such as journals (eg, Nature Portfolio^15^) and funding agencies, that require the sharing of code and, if possible, data. Reproducible coding practices also improve study validity and quality. Importantly, reproducibility and validity are not the same—a perfectly reproducible, but poorly designed study will not produce valid results, and vice versa.^16^ Reproducible practices simply make code less prone to errors and facilitate review of analysis, code and study design choices within the scientific community.^17 18^

Additionally, reproducible coding practices benefit individual researchers in multiple ways. Adopting reproducible coding practices enhances efficiency both for individual scholars and within research groups, because well-written code can be easily reused in new studies using similar data or methods. Sharing code and data openly further enhances impact, as the methodology is more likely to be used by other scholars. Preliminary findings from other disciplines, such as evolutionary biology, suggest that papers with shared code and data may accumulate citations faster.^19^ Ultimately, this culminates in a scientific landscape in which every paper includes reproducible, well-documented code—allowing researchers to directly build on prior work by integrating existing scripts into their own analyses.

## Recommendation 2: implement code review

Despite its widespread use in software engineering, systematic examination of code by peers during code review is not commonly implemented in medical research institutions. Code review benefits reproducibility, as it ensures adherence to coding standards and improves code quality.^17^ The code author will be more thorough when they know the code will be reviewed and the code reviewer can suggest improvements for transparency and comprehensibility of the code.^20^

In addition, code review improves research validity, because it helps to identify bugs and small errors and fosters a discussion on choices that were made during data analysis.

Individual researchers additionally benefit from code review by being exposed to new techniques that they might normally not use themselves. It also fosters further collaborations within a research group.^17^ However, it should be noted that code review is time-intensive, which is a significant barrier for its implementation.^17^ Offering the possibility for authorship could be a way to facilitate its implementation. Furthermore, including quality checks, such as unit tests^21^ in the code, makes code review easier. Examples are visualisations of data before and after preprocessing, checking the assumptions of statistical tests and providing unit tests for tailor-made functions. In online supplemental file 1, we provide examples of data visualisation (lines 265–280), unit tests (lines 170–204) and assumption checks (lines 326–380). Unit tests are automated checks to verify that individual parts of the code, such as functions or processing steps, perform as intended.

## Recommendation 3: write comprehensible code

A crucial aspect of reproducible research is comprehensibility, that is, the ease with which a third person can understand the structure, logic and functionality of analyses. Although less commonly noted as part of reproducibility, comprehensibility is essential as research that is not comprehensible to third parties cannot be adequately reproduced.^3^ There are multiple steps one can take to start improving the comprehensibility of code, which we noted in box 1. Overall, we recommend writing code in a way that clearly communicates its purpose and functionalities, ensuring clarity for code reviewers and future replication analyses. A first step is to use a clear structure, for example with adequate use of headings, a ‘ReadMe’ file explaining the workflow, and a data dictionary describing the variable names. Implementing loops and functions may further improve comprehensibility,^22^ because inefficient code that is extensive and repetitive can be hard to comprehend. Another step is to document the specific versions of used software and packages, because functionalities can change over time. Those with more advanced programming skills can take this one step further by applying ‘containerisation’, which includes saving the code and the corresponding computer settings as a whole.^4^

Box 1 **Examples of key elements to include in code to improve comprehensibility**

- **Structure.** A clear structure is important to help a third party maintain an overview when reading your analyses (eg, by including headings, see Supplement 1 line 18). A key component is to provide a ‘ReadMe’. This document provides an overview of the datasets that are used, the different analytical steps, and, if applicable, the different scripts that are used. Moreover, comprehensibility can be improved by using consistent and clear names for datasets, variables and other objects and by providing a data dictionary. In a data dictionary, one describes the variables in the dataset in more detail.
- **Efficiency.** Writing code that is efficient (ie, requires as little lines of code as necessary to complete a task) improves the comprehensibility of the code. Examples are making effective use of functions (Supplement 1, lines 137-169) and loops, if it does not compromise the code’s comprehensibility.
- **Documentation.** Documentation refers to all extra information that is provided in the code, which improves the comprehensibility. Examples are using comments to explain the purpose of a code section or making use of documentation tools, such as *Markdown* (see Supplement 1) or *Jupyter*. Additionally, it is important to document the version of software (eg, packages, Supplement 1 lines 47-111) and input data that is used.

## Recommendation 4: report decisions transparently

Another key part of reproducible research is being transparent about your research questions, design choices and analytical choices.^16^ This can be enhanced by writing a detailed analysis plan, which is shared or preregistered^3^ and by following reporting guidelines, such as the STROBE guidelines for observational studies in epidemiology.^23^ However, researchers continuously make decisions in daily research practice that were not detailed in the analysis plan or deviate from the plan.^24^ We recommend noting and reporting these decisions in the analytical code—analogous to log-keeping in laboratories—to improve reproducibility. Increasing transparency gives new researchers a realistic impression of research practices and the associated decisions that are made throughout the process.^4^

In box 2, we note several examples of steps in medical research that can require decision making and can be logged in the code. These include a clear description of steps taken in data preprocessing, cleaning and formatting, a detailed description of the sample selection, and reporting any deviations from the study protocol. Reporting on all these decisions in code is easiest when using executable documents such as *R Markdown* (see online supplemental file 1) or *Jupyter*. A more advanced method of logging decisions in daily research practice is using version control systems such as GIT (https://git-scm.com). These tools enable researchers to manage different versions of their files, collaborate with others and maintain a history of modifications. This way, one can revert to earlier versions if needed and clearly document the evolution of the analysis.

Box 2 **Reporting research decisions in code to improve transparency**

- **Data cleaning.** Before starting the statistical analyses, raw data should always be prepared by correcting or removing errors and formatting the data appropriately. To ensure reproducibility, it is crucial that all steps taken during data cleaning are transparently noted in the analytical code (Supplement 1 lines 112-263), so that a third person can accurately reproduce the analytical dataset from the raw dataset. Ideally, decisions are supported by data visualisations and a written rationale.
- **Sample selection.** The analytical sample is often selected from the full dataset based on in- and exclusion criteria, such as data availability, comorbidities or demographic factors. These steps should be described in such detail that the sample derivation can be independently reproduced (Supplement 1 lines 226-263).
- **Deviations from the study protocol.** Any deviations from the analysis plan, eg, data transformations, should be transparently reported (Supplement 1 lines 348-376). If any analyses are performed, but not reported in the published research article, a rationale for not reporting the findings should be provided in the code.

## Recommendation 5: focus on accessibility of code and data

Last, efforts taken to improve transparency or comprehensibility of code are fruitless if the analytical code and data are not made available.^1^ It may not always be possible to share analytical data, and when individual-level participant data is shared, researchers should be mindful that this is in compliance with participant consent and local data protection laws. They should take precautions to protect privacy and prevent re-identification, for example by masking quasi-identifiers and applying k-anonymity. However, even when data cannot be shared freely, sharing the corresponding code is still informative to get an overview of data management and the performed statistical analyses.^25^ For instance, providing an overview of the data cleaning process and the handling of missing data can help the reader to better understand the study, even without accessing the data. Moreover, researchers can consider sharing metadata such as variable types and labels, which facilitates reproducibility for future researchers working with the same dataset (eg, when working with the UK Biobank).^4^ This approach is in line with the *FAIR* guidelines proposing that data should be findable, accessible, interoperable and reusable.^26^ If data cannot be shared freely, authors can still state how the data can be accessed if permission is obtained.^1^

We identified and listed different options to share data and analytical code, each with their own advantages and disadvantages in table 1. Sharing code and data as a supplement to a paper is the most direct way of improving the reproducibility of a specific paper. However, it has the large disadvantage that many scientific journals do not facilitate the direct upload of code. An alternative is uploading the code and, if possible, data via a personal account on an open repository (eg, https://osf.io/; https://zenodo.org/) and sharing a DOI link in the publication. Due to strict data sharing policies, research institutions often have closed repositories, which protect data and can foster collaboration within the institution but severely limit accessibility. For example, the Rotterdam Study currently maintains a repository that is managed by a data management team, ensuring systematic organisation and maintenance. Based on these observations, we tentatively conclude that an openly accessible repository that is hosted and facilitated by the research institution is the way forward, because it fosters collaboration and efficiency within a research group while making the resources accessible for the wider scientific community. An example is the OxCovid19 Database, which collects, curates and openly shares global COVID-19 data.^27^

**Table 1:** Possibilities to share data and analytical code

	Supplement	Public repository (individual use)	Institutional repository (open)	Institutional repository (closed)
**How?**	Add data and supplementary analyses as an online supplement to a publication	Share code and data via a personal account on a repository, such as GitHub or Zenodo	Share code and data on an online platform maintained by an institution or research group	Code and data are archived locally by the institution and can only be accessed on request
**Findable?**				
Readers of paper	Yes	Yes, if a DOI is provided in the paper	Yes, if a DOI is provided in the paper	Not accessible
Researchers working with the same data	Yes	Yes	Yes	Yes
Wider scientific public	Yes	Yes	Yes	Not accessible
**Required resources**	Part of the usual writing process. No extra resources needed	Easy to set up and manage, without need for support	Institutional maintenance, for example, requesting materials after publication	Institutional maintenance, for example, requesting materials after publication
**Can materials be updated?**	No	Yes	Yes, but might require data manager	Yes, but might require data manager
**Further considerations**	Sharing code in a supplement may be difficult due to the common formatting of the supplement (ie, Excel or Word files)	Some repositories (eg, GitHub) facilitate version control	Facilitates efficient research and new collaborations for researchers working with the same datasets (eg, a cohort study)	Suitable when strict data sharing policies apply

## Code checklist

**Figure 1:**
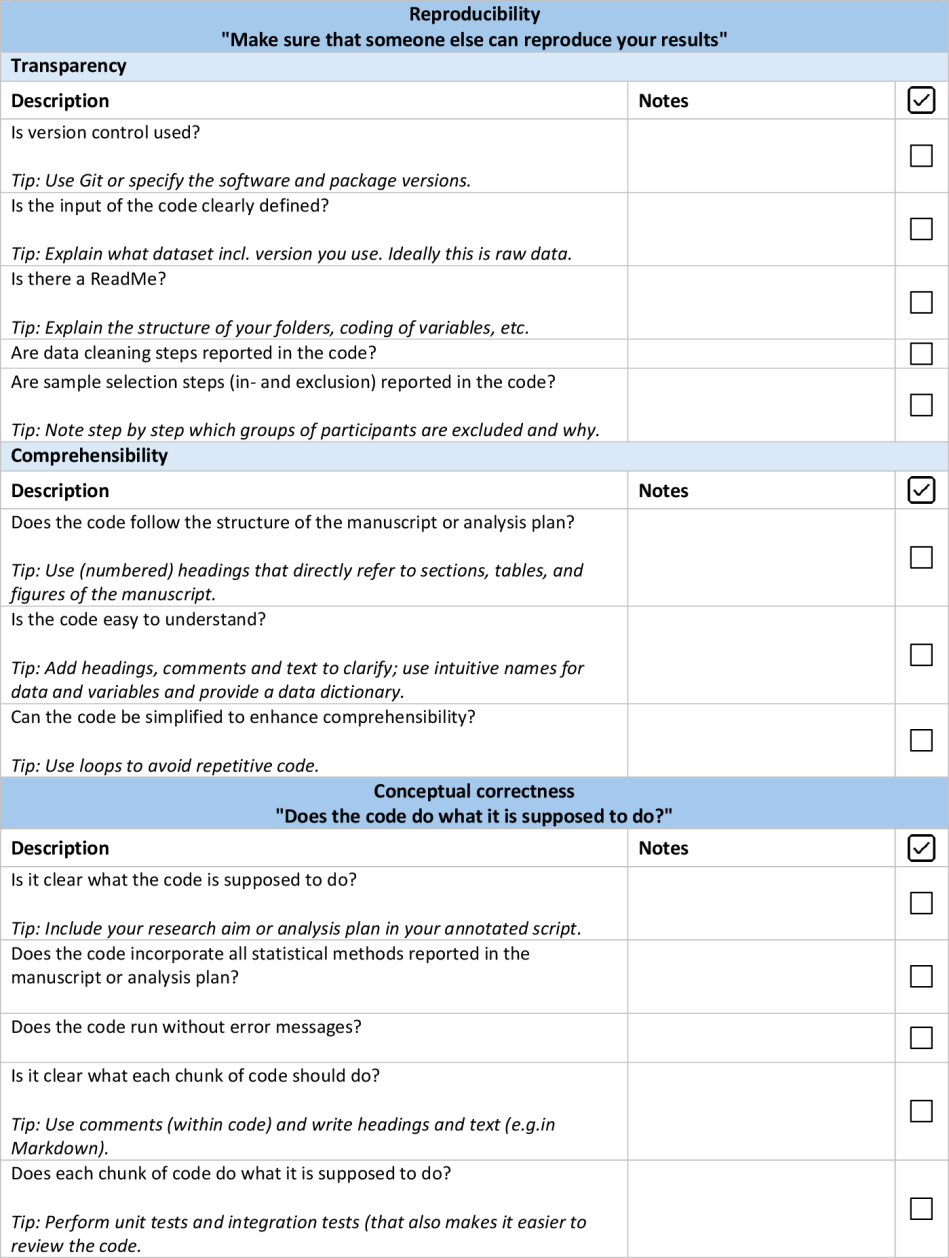
Code review checklist to assess reproducibility and conceptual correctness of code used for data preprocessing, dataset generation and statistical analyses. The checklist is structured into two overarching domains: reproducibility (top section), which includes the subdomains transparency and comprehensibility and conceptual correctness (bottom section). Each domain contains descriptions (left column) with practical tips for implementation (italic text) and corresponding columns for reviewer notes and completion check marks.

Based on the recommendations outlined before, we developed a code checklist (figure 1) aimed at facilitating the implementation of code review. This checklist can be used during code review and serves two separate aims, which are reflected in its two parts. First, the checklist is used to assess the reproducibility of the code, focusing on our recommendations for writing comprehensible and transparent code. Second, the checklist provides guiding questions that can be used to assess the conceptual correctness of the code (ie, does the code do what it is supposed to do?). Here, the code reviewer focuses on whether the code performs the analyses that are outlined in the accompanying statistical analysis plan or paper in the correct way. The checklist contains practical tips to improve code and leaves room for comments that arise during the review process. Additionally, one can use the code checklist to perform a structured self-review, but this approach is more likely to overlook reproducibility issues due to the reviewer’s familiarity with the code’s logic and environment.

## Discussion

Reproducibility is essential for ensuring the integrity, reliability and advancement of research. We reviewed current coding practices within a population-based cohort study and outlined five actionable steps that medical researchers can take to improve reproducibility. These recommendations provide medical researchers with a starting point to write reproducible code, while guidance for more advanced steps, such as containerisation^28^ or advanced version control,^29^ can be found elsewhere.

While reproducible code is a crucial part of reproducible medical research, it should be implemented in conjunction with reproducible documentation of research methods in an analysis plan and manuscript. More detailed recommendations for reproducible reporting of medical research are provided elsewhere, especially within the EQUATOR framework (https://www.equator-network.org/).^3 4^ Critical components in the documentation of statistical methods include detailed descriptions of variable selection processes, the statistical tests including assumption checks, potential sensitivity analyses and the methods used for data presentation.^30^ Detailed reporting of statistical methods may be further enhanced by using a corresponding checklist (eg, Dwivedi and Shukla^31^).

Importantly, efforts of individual researchers to improve reproducibility will have a wider impact if they are supported by research institutions, funding agencies and publishers. In line with McDermott and colleagues,^32^ we propose that these parties should have an accessibility policy that supports the sharing of data and code as much as possible. Publishers should facilitate and encourage the upload of code at the submission stage or facilitate easy linkage to public repositories such as *ZENODO*. Funding agencies can support reproducibility by allocating funding specifically to the dissemination of code and data or the implementation of code review, acknowledging that this will increase research quality and output downstream. For example, funding calls could explicitly include budget lines for reproducibility infrastructure.

The recommendations in this work are based on our review of the archive of the Rotterdam Study. Although we are not aware of existing archives with a similar set-up and length of follow-up, future work could expand our recommendations by surveying coding practices in other medical research contexts, for example, from clinical trials. There is also a need for systematic evidence on the effectiveness of practices such as code review in improving research reproducibility and quality. Ultimately, we believe that all steps, even small ones, taken towards reproducibility contribute towards a research environment where reproducibility and thorough peer review are standard practices.

## Supplementary material

10.1136/bmjopen-2025-104691 online supplemental file 1
