# Supplementary material for: Improving reproducibility of data analysis and code in medical research: 5 recommendations to get started
Source: BMJ Open. 2025 Oct 2;15(10):e104691. doi: 10.1136/bmjopen-2025-104691 (PMC12496075; doi:10.1136/bmjopen-2025-104691)
Supplement: online supplemental file 1 [file bmjopen-15-10-s001.pdf]

# Reproducible Coding: A Practical Example

A.M. Streiber & S.J.W Hoepel

Last modified: 24-07-2025

## Proposed Research

### Background

This is a hypothetical study designed solely for educational purposes. The research question presented is not based on an actual research project but is intended to illustrate how medical research analyses can be conducted in a transparent and reproducible manner.

The accompanying R script clearly specifies which elements are included in a hypothetical manuscript or analysis plan and uses consistent code labels that correspond to the names of tables and figures. We recommend this practice, as it facilitates reproducibility and improves comprehensibility.

For demonstration purposes, we aim to answer the following research question:

- Is systolic blood pressure associated with age in people 40 years and older, when controlling for sex and ethnicity?

### Methods

For this illustrative analysis, we used data from the National Health and Nutrition Examination Survey (NHANES), accessed via the R package `nhanesA`. NHANES is a nationally representative cross-sectional survey conducted by the National Center for Health Statistics (NCHS), designed to assess the health and nutritional status of adults and children in the United States. Detailed documentation on the survey design, variables, and sampling procedures is available on the NCHS website.

The aim of this example is to demonstrate how to conduct a transparent and reproducible analysis pipeline. The analysis includes the following steps:

- **Data acquisition and cleaning**
  We used the `nhanesA` package to download relevant datasets, merged them as needed, and applied basic data cleaning procedures. These included removing missing values, restricting the sample to participants aged ≥ 40 years, and ensuring appropriate coding of categorical variables (e.g., sex, ethnicity).

- **Exploratory data analysis**
  We visualized the distribution of systolic blood pressure, sex, and covariables using histograms and bar graphs.

- **Descriptive statistics**
  We produced a descriptive overview of the sample, including means and standard deviations for continuous variables and frequencies and percentages for categorical variables. These are presented in a summary table.

- **Statistical analysis**
  To address our educational research question — *Is systolic blood pressure associated with age in people 40 years and older, when controlling for sex and ethnicity?* — we fitted a linear regression model with systolic blood pressure as the outcome and age in years as the primary determinant. Sex and ethnicity were included as covariates. We checked key assumptions of linear regression, including normality of residuals and homoscedasticity.

# 0. Data Preparation

## 0.1 Acquire Packages

```
library(nhanesA)      # Accessing the data used in this example
library(rstudioapi)   # version control
library(dplyr)        # data transformation and presentation
library(ggplot2)      # plotting data
library(table1)       # creating descriptive tables
library(testthat)     # performing unit tests
```

## 0.2 Version control

Below, you can see the R, R studio, and package versions that have been used in this project.

```
## R version 4.2.1 (2022-06-23)
## RStudio Version: 2022.12.0.353 ( desktop )

## [1] "base 4.2.1"
## [1] "brio 1.1.3"
## [1] "cli 3.6.1"
## [1] "colorspace 2.0.3"
## [1] "compiler 4.2.1"
## [1] "datasets 4.2.1"
## [1] "digest 0.6.30"
## [1] "dplyr 1.1.1"
## [1] "evaluate 1.0.3"
## [1] "fansi 1.0.3"
## [1] "fastmap 1.1.1"
## [1] "foreign 0.8.82"
## [1] "Formula 1.2.4"
```

```
## [1] "generics 0.1.3"
## [1] "ggplot2 3.4.2"
## [1] "glue 1.6.2"
## [1] "graphics 4.2.1"
## [1] "grDevices 4.2.1"
## [1] "grid 4.2.1"
## [1] "gtable 0.3.6"
## [1] "htmltools 0.5.7"
## [1] "httr 1.4.5"
## [1] "knitr 1.40"
## [1] "lifecycle 1.0.3"
## [1] "magrittr 2.0.3"
## [1] "methods 4.2.1"
## [1] "munsell 0.5.0"
## [1] "nhanesA 1.3"
## [1] "pillar 1.8.1"
## [1] "pkgconfig 2.0.3"
## [1] "plyr 1.8.8"
## [1] "R6 2.5.1"
## [1] "Rcpp 1.0.9"
## [1] "rlang 1.1.0"
## [1] "rmarkdown 2.17"
## [1] "rstudioapi 0.17.1"
## [1] "rvest 1.0.3"
## [1] "scales 1.2.1"
## [1] "stats 4.2.1"
## [1] "stringi 1.7.8"
## [1] "stringr 1.5.0"
## [1] "table1 1.4.3"
## [1] "testthat 3.1.7"
## [1] "tibble 3.2.1"
## [1] "tidyselect 1.2.0"
## [1] "tools 4.2.1"
## [1] "utf8 1.2.2"
## [1] "utils 4.2.1"
## [1] "vctrs 0.6.1"
## [1] "withr 3.0.2"
## [1] "xfun 0.37"
## [1] "xml2 1.3.3"
## [1] "yaml 2.3.6"
```

## 0.3 Data Acquisition and Data Cleaning

For this practical example, we used NHANES data collected between 2011 and 2012. NHANES collects data annually, and we randomly chose the data from the 2011-2012 cycle. The suffix `_G` indicates that our data are from that round.

```
# Demographic data from the 2011-2012 cycle
demographics <- nhanes("DEMO_G")
# Blood pressure data from the 2011-2012 cycle
```

```
blood_pressure <- nhanes("BPX_G")

# Merge the data together into one dataset
analysis_data <- merge(x=demographics, y=blood_pressure, by = "SEQN", all = T
RUE)
rm(demographics, blood_pressure)

# Check number of variables (i.e. columns)
ncol(analysis_data) # 74

## [1] 74
```

Currently, there are 74 variables in the dataset. However, we are only interested in a subset of variables, namely age, sex, ethnicity, and systolic blood pressure. Systolic blood pressure has been measured consecutively three times. If one of the measures was interrupted or incomplete, a fourth attempt was performed.

We want to use the average score across the systolic blood pressure values, but only when blood pressure was measured at least twice. We wrote a function that accounts for missing data and does not calculate the mean if there are more than two missing values for blood pressure.

```
## this function takes as input:
# df; the dataframe containing the data
# columnames; the names of the relevant columns, as a vector
# max_missing: the maximum number of missing values, with 2 as default

compute_mean_sbp <- function(df, column_names, max_missing = 2) {

  # Check if the required columns are present in the dataset
  missing_cols <- setdiff(column_names, names(df))
  if (length(missing_cols) > 0) {
    stop("Missing expected BP columns: ", paste(missing_cols, collapse = ", "
))
  }

  # Calculate for each row the number of missing values in these columns
  df$missing_obs <- rowSums(is.na(df[, column_names]))

  # create an empty column for average blood pressure values
  df$mean_sbp <- NA

  for(i in 1:nrow(df)){

    if(df[i, "missing_obs"] > max_missing){   ##if more observations are miss
ing than the set max
      df[i, "mean_sbp"] <- NA    }  ## the mean is set to NA

    else{ ## otherwise the mean is calculated
```

```
      df[i, "mean_sbp"] <- rowMeans(df[i, column_names], na.rm = TRUE)
    }
  }

  return(df)
}
```

Using the `test_that()` function, we performed a unit test to make sure that the function runs as intended. For this we first create a simulated dataframe called `test_df`. In this test data frame, we introduced different patterns of missing data and specified which mean values we expect per row (see `# Expected values`). The unit test checks if the means computed with the function match with the expected means using the `expect equal()` function.

Unit tests are essential for ensuring that functions perform as expected under various conditions.

```
test_that("compute_mean_sbp calculates correct means", {
  # Create simulated data with different missingness patterns
  test_df <- data.frame(
    Col1 = c(120, NA, 130, NA),
    Col2 = c(NA, 140, NA, NA),
    Col3 = c(NA, 145, 135, NA),
    Col4 = c(NA, 125, NA, NA)
  )

  result_df <- compute_mean_sbp(test_df, column_names = c("Col1", "Col2", "Co
l3", "Col4"))

  # Expected values
  expected_means <- c(
    NA,                                # in the first row, 3 values are
missing, so we expect NA
    mean(c(140, 145, 125), na.rm = TRUE),   # in the second row 3 values are
reported, so we expect their mean
    mean(c(130, 135), na.rm = TRUE),        # in the third row 2 values are r
eported, so we expect their mean
   NA                                  # in the fourth row, 4 values are
missing, so we expect NA
  )

  expect_equal(result_df$mean_sbp, expected_means)
}) # passed

## Test passed 😀
```

Now that we know that the function works, we can apply it to our data frame.

```
analysis_data <- compute_mean_sbp(analysis_data, column_names = c("BPXSY1", "
BPXSY2", "BPXSY3", "BPXSY4"))
```

Next, we select the data that we need for our analyses and assign more comprehensible variable names. The data set includes two variables on ethnicity. We decided to use the more detailed variable `RIDRETH3`, which provides information on 7 different ethnicity categories including non-hispanic Asian. The variable is coded as follows:

1 = Mexican American (reference group)

2 = Other Hispanic

3 = Non-Hispanic White

4 = Non-Hispanic Black

6 = Non-Hispanic Asian

7 = Other ethnicity - Including Multi-Ethnic

```
analysis_data <- analysis_data |>
  # Rename data
  rename(participant_id = SEQN,
         age = RIDAGEYR,
         sex = RIAGENDR,
         ethnicity = RIDRETH3) |>
  # Select variables you need
  select(participant_id, age, sex, ethnicity, mean_sbp)
```

Finally, we remove all participants with missing systolic blood pressure data and individuals who are younger than 40. We keep track of the number of participants we exclude to compute a flow chart

```
# Total number of participants
n_total <- nrow(analysis_data) # 9756

# Remove participants with missing blood pressure data
analysis_data <- analysis_data |> na.omit(mean_sbp)
n_complete_sbp <- nrow(analysis_data) # 7018
# Check if all missing blood pressure data were successfully removed
sum(is.na(analysis_data$mean_sbp)) # 0; all missing data removed

## [1] 0

# Remove everyone younger than 40
analysis_data <- analysis_data |> filter(age >= 40)
n_at_least_40 <- nrow(analysis_data) # 3277
# Check if it worked
summary(analysis_data$age) # Yes; Minimum age = 40

##    Min. 1st Qu.  Median    Mean 3rd Qu.    Max.
##   40.00   49.00   59.00   59.42   69.00   80.00
```

- **9756** participants of the 2011-2012 round have available data.

- Out of all participants with available data, **7018** participants have at least 2 available systolic blood pressure measures.
- Out of these participants, 3277 participants are at least 40 years old. Thus, the final sample is comprised of **3277** participants.

```
# Final check if all variables are coded correctly (i.e. numeric values = num
eric and factors = factor)
str(analysis_data) # Yes
```

```
## 'data.frame':    3277 obs. of  5 variables:
##  $ participant_id: num  62164 62172 62174 62177 62178 ...
##  $ age           : num  44 43 80 51 80 55 70 57 42 62 ...
##  $ sex           : Factor w/ 2 levels "Male","Female": 2 2 1 1 1 1 1 1 1 2
...
##  $ ethnicity     : Factor w/ 6 levels "Mexican American",..: 3 4 3 5 3 5 4
3 5 1 ...
##  $ mean_sbp      : num  118 102 98 147 122 ...
##  - attr(*, "na.action")= 'omit' Named int [1:2738] 2 7 8 13 15 22 23 28 36
38 ...
##   ..- attr(*, "names")= chr [1:2738] "2" "7" "8" "13" ...
```

# 1. Data exploration

## 1.1 Data distribution

```
# Histogram age
hist(analysis_data$age)
```

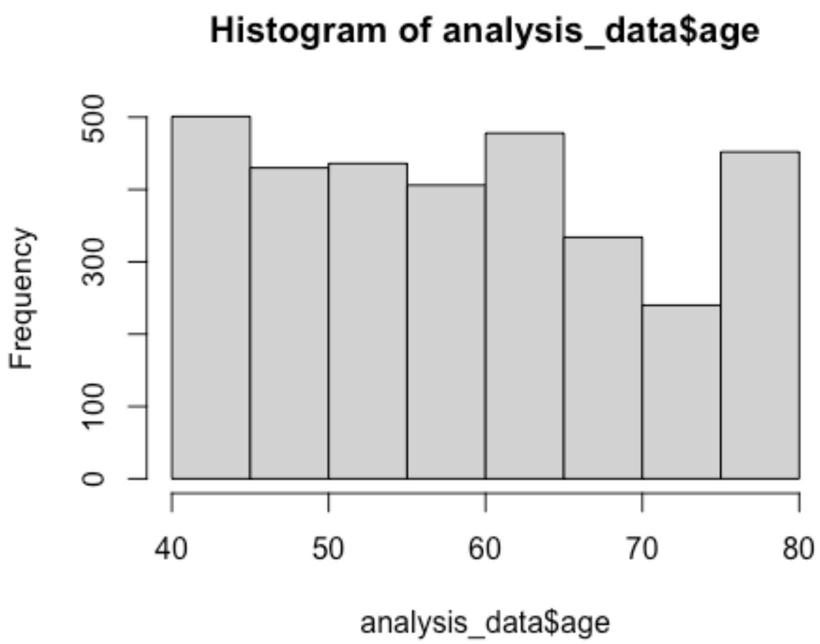

```r
# Histogram systolic blood pressure
hist(analysis_data$mean_sbp)
```

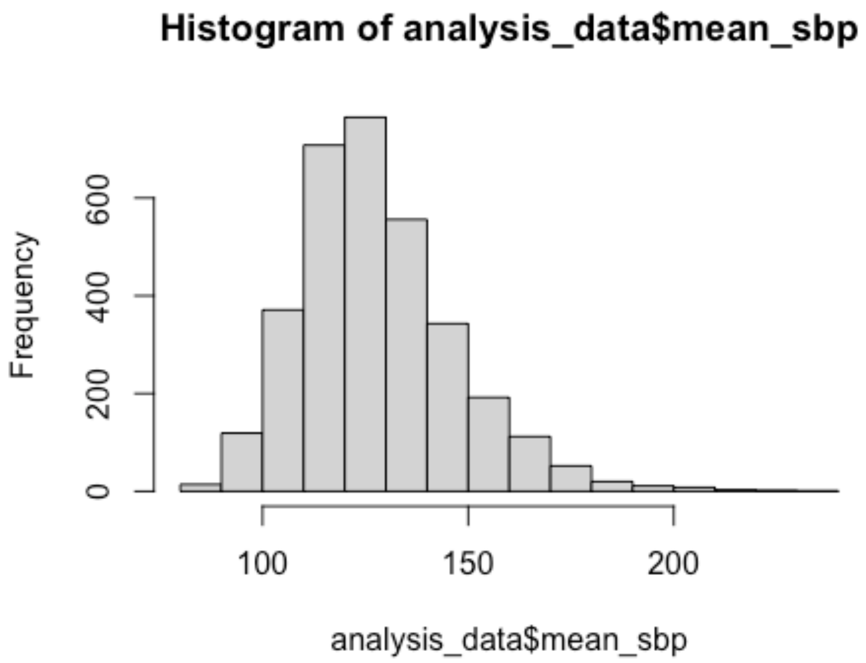


```r
# Bar graph sex
ggplot(analysis_data, aes(sex)) + geom_bar()
```

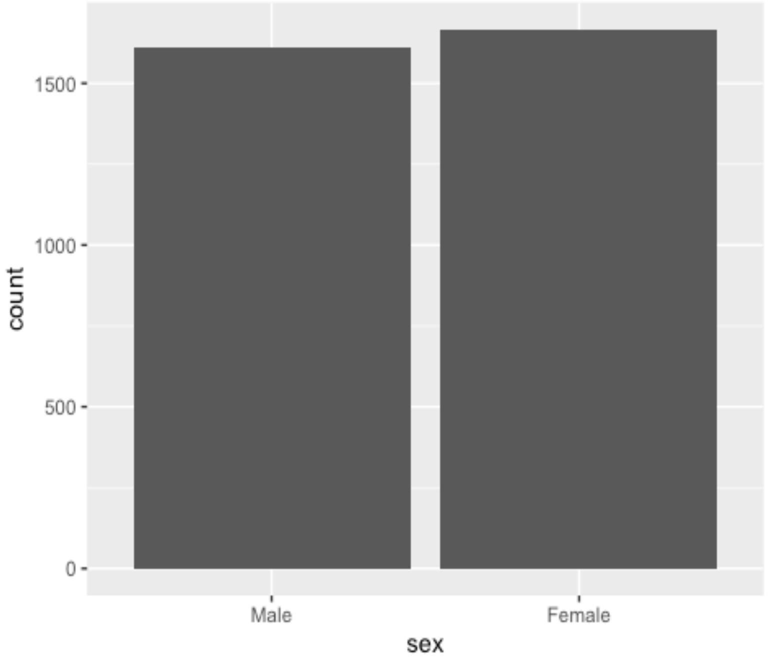

```r
# Bar graph ethnicity
ggplot(analysis_data, aes(ethnicity)) + geom_bar()
```

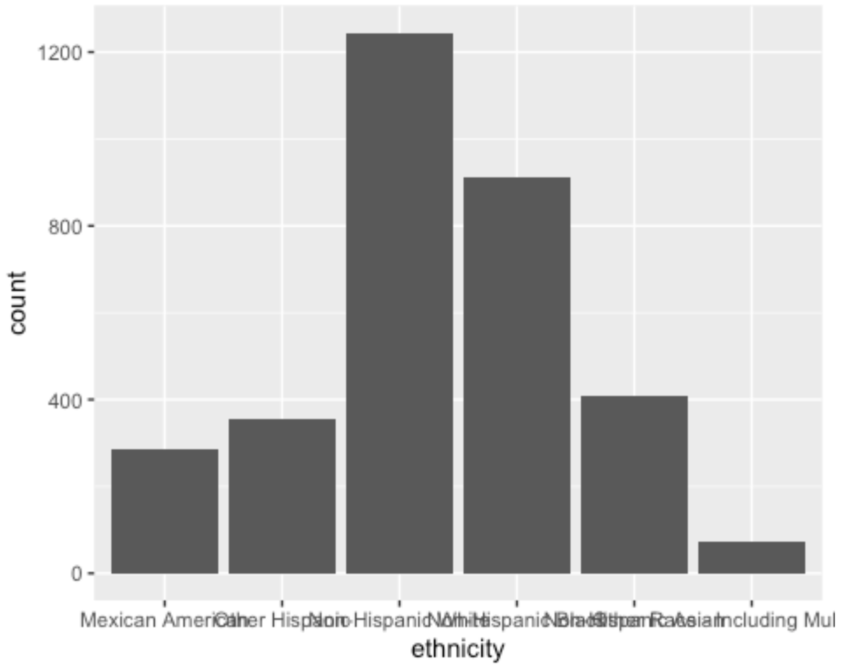


## 1.2 Scatterplot age and systolic blood pressure

```r
ggplot(analysis_data, aes(age,mean_sbp)) + geom_point()
```

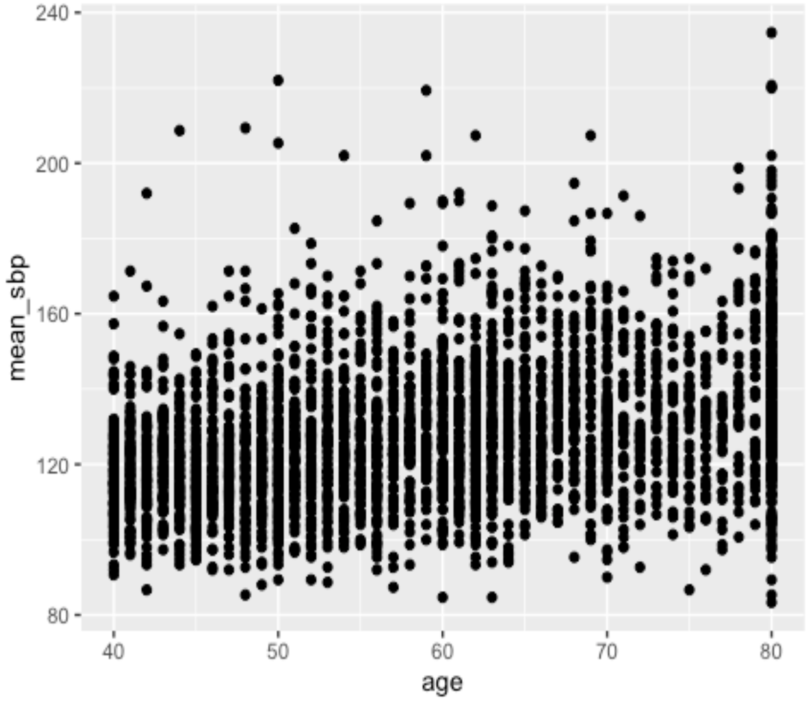

# 2. Data Analyses

## 2.1 Descriptives

### 2.1.1 Creation Table 1

```
# Create labels for your variables to be displayed in the table
labels <- list(
  variables=list(age = "Age in years",
                 sex = "Sex",
                 ethnicity = "Ethnicity",
                 mean_sbp = "Mean systolic blood pressure"))

# Compile dataset into a list
descriptives_list <- c(list(Total=analysis_data))

table_one <- table1(descriptives_list,labels=labels,
                    caption= "Baseline Characteristics",
                    footnote = "Continuous values are reported as mean (SD) a
nd median (min, max). Categorical variables are reported as n(%)")
```

### 2.1.2 Output Table 1

```
## Get nicer `table1` .docx output by simply installing the `flextable` packa
ge

##                                                         Total
## 1                                                    (N=3277)
## 2                          Age in years
## 3                             Mean (SD)         59.4 (12.1)
## 4                     Median [Min, Max] 59.0 [40.0, 80.0]
## 5                                   Sex
## 6                                  Male        1609 (49.1%)
## 7                                Female        1668 (50.9%)
## 8                             Ethnicity
## 9                      Mexican American          284 (8.7%)
## 10                       Other Hispanic         357 (10.9%)
## 11                   Non-Hispanic White        1245 (38.0%)
## 12                   Non-Hispanic Black         910 (27.8%)
## 13                   Non-Hispanic Asian         407 (12.4%)
## 14   Other Race - Including Multi-Racial          74 (2.3%)
## 15         Mean systolic blood pressure
## 16                            Mean (SD)          128 (19.2)
## 17                    Median [Min, Max]   126 [83.3, 235]
```

## 2.2 Regression analysis

### 2.2.1 Model building

Research question: Is systolic blood pressure associated with age in people 40 years and older, when controlling for sex and ethnicity?

We use linear regression, with systolic blood pressure as the dependent variable and age, sex, and ethnicity as the independent variables.

```
regression <- lm(mean_sbp ~ age + sex + ethnicity, data = analysis_data)
```

### 2.2.2 Assumption Check

Before we have a look at the results, we need to check if the assumptions underlying the linear regression model are fulfilled. We will check that using the `plot()` function.

```
# Linearity
plot(regression, 1)
```

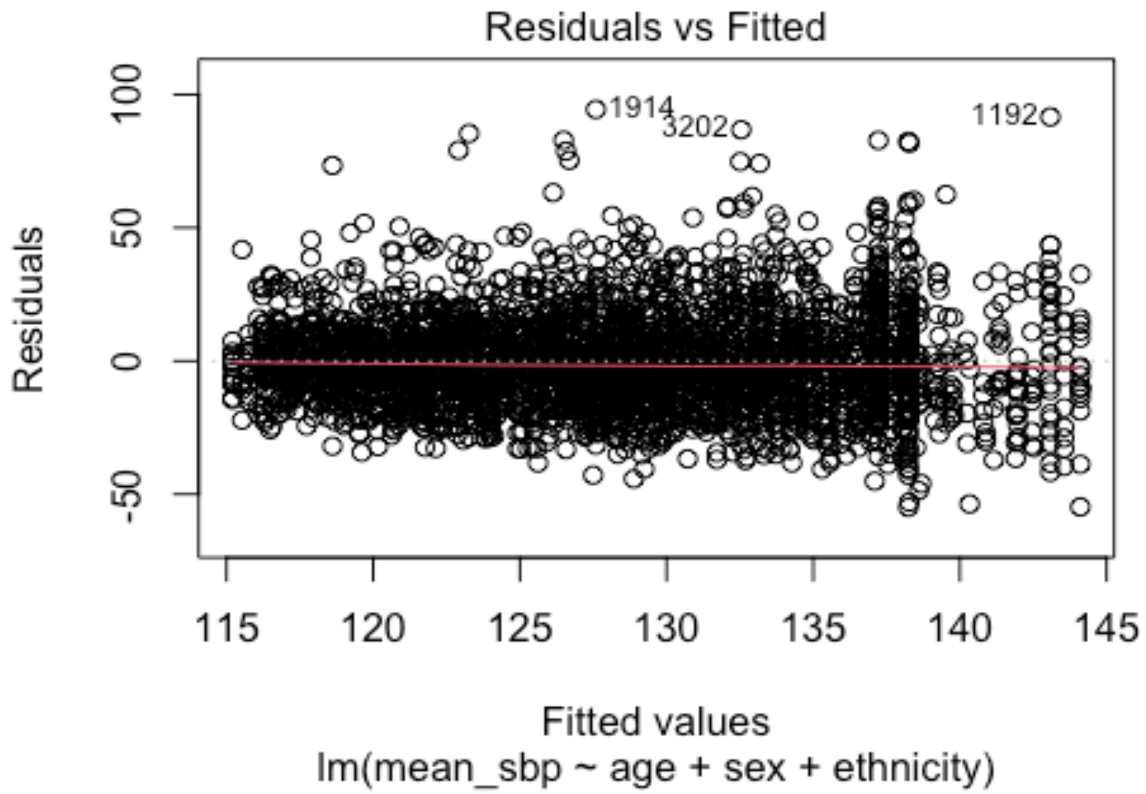


The plot indicates *no violation* of the linearity assumption.

- We conclude that the linearity assumption is met.

```r
# Homoscedasticity
plot(regression, 3)
```

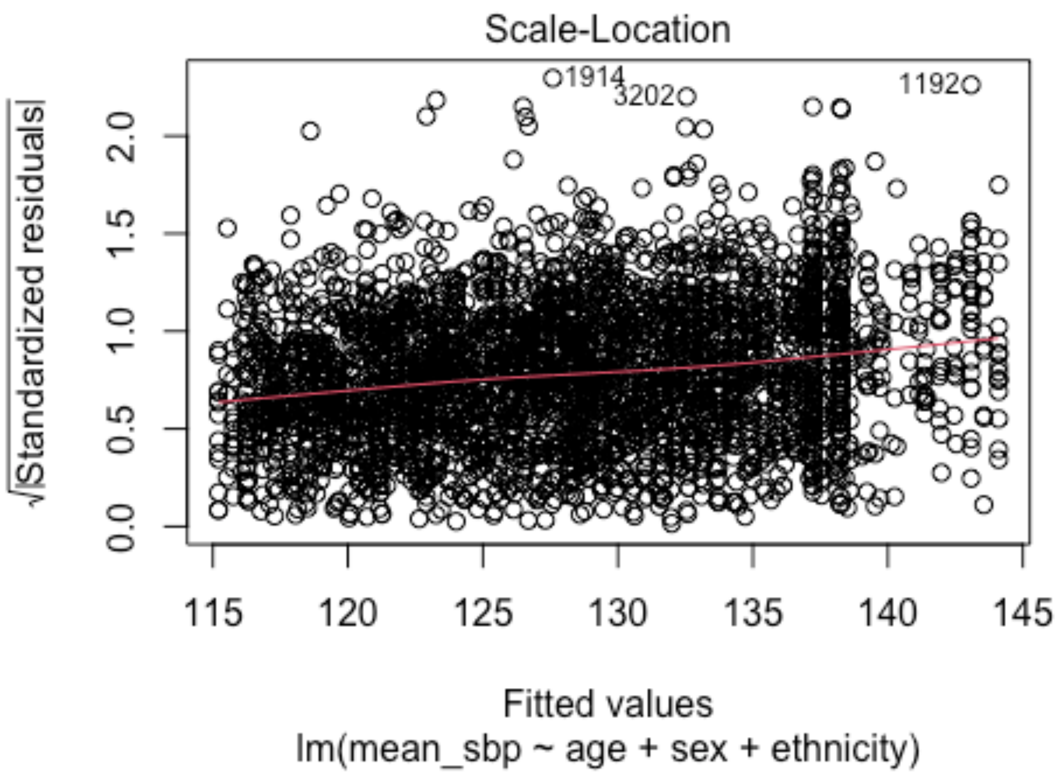


The plot indicates *no severe violation* of the homoscedasticity assumption. The spread of residuals appears fairly constant across the range of fitted values, with no clear pattern or funnel shape. The variability of the residual points increases slightly with the value of the fitted outcome variable (indicated by the red line). Hence, we observe some heteroscedasticity.

A possible solution to reduce heteroscedasticity is to log or square root transform the outcome variable (mean systolic blood pressure). We will apply the log transformation and see if it improves homoscedasticity.

```r
# Log-transform the data
regression_log <- lm(log(mean_sbp) ~ age + sex + ethnicity, data = analysis_d
ata)

# Check homoscedasticity using log-transformed data
plot(regression_log, 3)
```

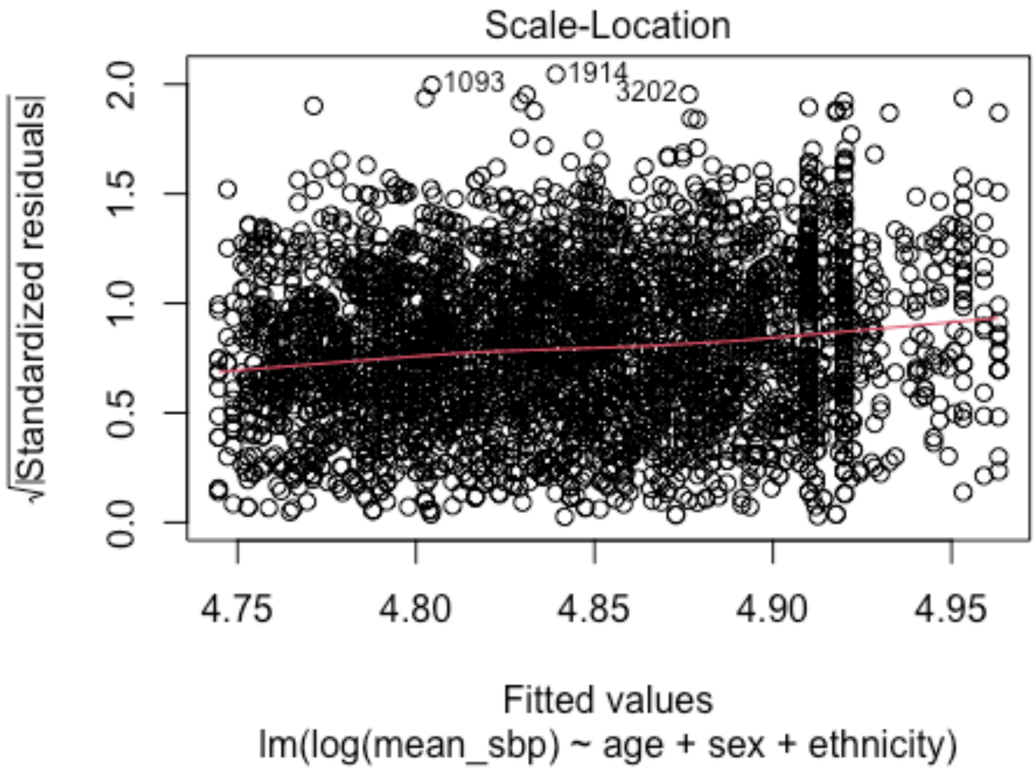


The log-transformation does not significantly improve homoscedasticity and, hence, provides no additional benefit in terms of meeting the regression assumptions.

- We conclude that the raw data adequately meet the homoscedasticity assumption.

```
# Normality of residuals
plot(regression, 2)
```

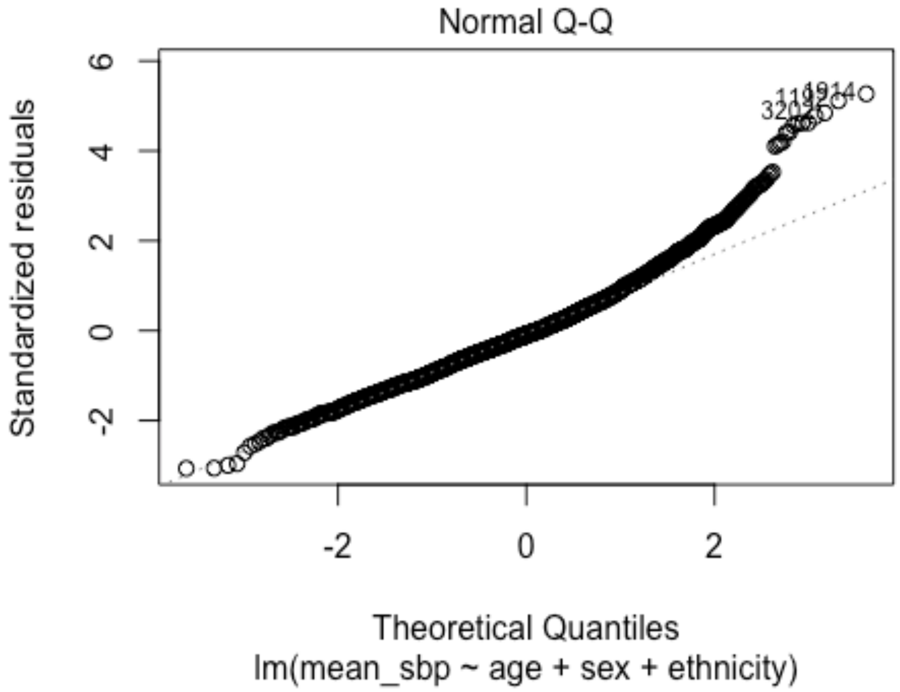

The data points mostly align along the reference line, suggesting that the residuals are *approximately normally distributed*. However, there are some deviations at both ends, particularly in the upper tail, where several points (e.g., 1149, 1919) deviate significantly from the line.

We will check if a log-transformation will help improve normality.

```
# Check normality of residuals using log-transformed data
plot(regression_log, 2)
```

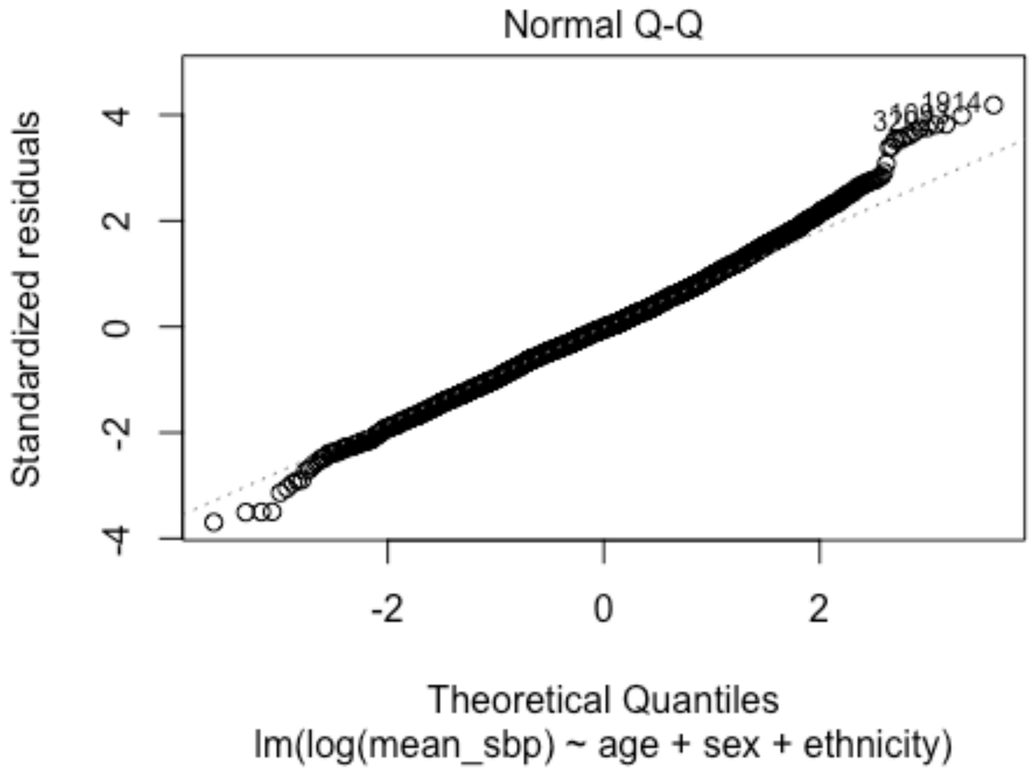


Following log-transformation, there are still deviations in the upper tail of the distribution.

- We conclude that the normality assumption is somewhat violated. However, given our large sample of > 3000 participants, we assume that the Central Limit Theorem mitigates the impact of non-normal residuals.

Overall, the hypotheses underlying linear regression analyses are sufficiently met. Since log-transformation of the outcome variable did not significantly improve normality and homoscedasticity and complicates the interpretation of results, we decided to proceed with the non-transformed systolic blood pressure data.

### 2.2.3 Output Table 2

First, we print the output of the linear regression model.

```
##
## Call:
## lm(formula = mean_sbp ~ age + sex + ethnicity, data = analysis_data)
##
## Residuals:
##     Min      1Q  Median      3Q     Max
## -54.908 -11.445  -1.575   9.684  94.407
##
## Coefficients:
##                                             Estimate Std. Error t value
## (Intercept)                                 95.49795    1.83515  52.038
## age                                          0.55037    0.02628  20.941
## sexFemale                                   -1.02086    0.62717  -1.628
## ethnicityOther Hispanic                     -0.23033    1.42934  -0.161
## ethnicityNon-Hispanic White                 -1.28568    1.19022  -1.080
## ethnicityNon-Hispanic Black                  4.57640    1.22181   3.746
## ethnicityNon-Hispanic Asian                 -0.96104    1.38675  -0.693
## ethnicityOther Race - Including Multi-Racial  1.62734    2.34034   0.695
##                                             Pr(>|t|)
## (Intercept)                                  < 2e-16 ***
## age                                          < 2e-16 ***
## sexFemale                                   0.103679
## ethnicityOther Hispanic                     0.871988
## ethnicityNon-Hispanic White                 0.280128
## ethnicityNon-Hispanic Black                 0.000183 ***
## ethnicityNon-Hispanic Asian                 0.488346
## ethnicityOther Race - Including Multi-Racial 0.486889
## ---
## Signif. codes:  0 '***' 0.001 '**' 0.01 '*' 0.05 '.' 0.1 ' ' 1
##
## Residual standard error: 17.93 on 3269 degrees of freedom
## Multiple R-squared:  0.1321, Adjusted R-squared:  0.1303
## F-statistic: 71.09 on 7 and 3269 DF,  p-value: < 2.2e-16
```

In the output, a p-value < 0.001 is noted for age, which indicates that in this sample of adults ≥ 40 years old, higher age is associated with higher mean systolic blood pressure.

Next, we print the effect size of age (i.e. the model coefficient) with the corresponding 95% Confidence Interval.

```
# Model Coefficient Age
round(mod_summary$coefficients[2, ], 3)
```

```
##   Estimate Std. Error    t value   Pr(>|t|)
##      0.550      0.026     20.941      0.000
```

```
# 95% Confidence Interval Age
round(confint(regression), 3)[2,]
```

```
##  2.5 % 97.5 %
##  0.499  0.602
```

We can interpret this coefficient in the following way: Per one year increase in age, systolic blood pressure will increase by 0.550 (95% CI: 0.499-0.602) units, given that all other covariates remain stable.

## 2.3 Sensitivity Analysis

Extreme values may disproportionately influence the results. Consequently, we have excluded all `mean_sbp` observations that were more than three standard deviations above or below the mean and performed the regression analyses in the less extreme subgroup.

```
# Exclude outliers in systolic blood pressure
# Compute mean
sbp_mean <- mean(analysis_data$mean_sbp, na.rm = TRUE)
# Compute standard deviation
sbp_sd <- sd(analysis_data$mean_sbp, na.rm = TRUE)

# Keep only values within 3 SD of the mean
sensitivity_data <- subset(analysis_data,
                           mean_sbp > (sbp_mean - 3 * sbp_sd) &
                           mean_sbp < (sbp_mean + 3 * sbp_sd))

# Check how many extreme observations have been removed
nrow(analysis_data) - nrow(sensitivity_data)
```

```
## [1] 37
```

```
# Re-run the regression on the restricted dataset
regression_sensitivity <- lm(mean_sbp ~ age + sex + ethnicity,
                             data = sensitivity_data)
```

```
##
## Call:
## lm(formula = mean_sbp ~ age + sex + ethnicity, data = sensitivity_data)
##
## Residuals:
##     Min      1Q  Median      3Q     Max
## -53.591 -10.741  -1.234   9.855  55.429
##
## Coefficients:
##                                             Estimate Std. Error t value
## (Intercept)                                 96.91454    1.70330  56.898
## age                                          0.50987    0.02443  20.867
## sexFemale                                   -1.21026    0.58140  -2.082
## ethnicityOther Hispanic                      0.62126    1.32388   0.469
## ethnicityNon-Hispanic White                 -0.78017    1.10482  -0.706
## ethnicityNon-Hispanic Black                  4.31985    1.13519   3.805
## ethnicityNon-Hispanic Asian                 -0.16455    1.28455  -0.128
## ethnicityOther Race - Including Multi-Racial  2.60760    2.16097   1.207
##                                             Pr(>|t|)
## (Intercept)                                  < 2e-16 ***
```

```
## age                                                < 2e-16 ***
## sexFemale                                          0.037455 *
## ethnicityOther Hispanic                            0.638906
## ethnicityNon-Hispanic White                        0.480145
## ethnicityNon-Hispanic Black                        0.000144 ***
## ethnicityNon-Hispanic Asian                        0.898076
## ethnicityOther Race - Including Multi-Racial 0.227643
## ---
## Signif. codes:  0 '***' 0.001 '**' 0.01 '*' 0.05 '.' 0.1 ' ' 1
##
## Residual standard error: 16.53 on 3232 degrees of freedom
## Multiple R-squared:  0.1314, Adjusted R-squared:  0.1295
## F-statistic: 69.84 on 7 and 3232 DF,  p-value: < 2.2e-16
```

```
# Model Coefficient Age
round(mod_summary_sensitivity$coefficients[2, ], 3)
```

```
##   Estimate Std. Error    t value   Pr(>|t|)
##      0.510      0.024     20.867      0.000
```

```
# 95% Confidence Interval Age
round(confint(regression_sensitivity), 3)[2,]
```

```
##  2.5 % 97.5 %
##  0.462  0.558
```

Per one year increase in age, systolic blood pressure will increase by 0.510 (95% CI: 0.462-0.558) units, given that all other covariates remain stable. These results are in line with the findings from our main analysis in 2.2. Therefore, we conclude that extreme observation did not influence our results. str

## 2.4 Exploratory Analysis

We explored sex-stratified models to see if the relationship between age and systolic blood pressure differs between men and women, as blood pressure patterns can vary by sex due to biological and lifestyle factors.

```
# Check levels of the sex variable
levels(analysis_data$sex)
```

```
## [1] "Male"   "Female"
```

```
# Subset and run models separately for males and females
regression_male <- lm(mean_sbp ~ age + ethnicity,
                      data = subset(analysis_data, sex == "Male"))

regression_female <- lm(mean_sbp ~ age + ethnicity,
                        data = subset(analysis_data, sex == "Female"))
```

```
##
## Call:
```

```
## lm(formula = mean_sbp ~ age + ethnicity, data = subset(analysis_data, 
##     sex == "Male"))
## 
## Residuals:
##     Min      1Q  Median      3Q     Max 
## -52.095 -11.175  -1.790   9.756  92.633 
## 
## Coefficients:
##                                                Estimate Std. Error t value
## (Intercept)                                   105.05612    2.51886  41.708
## age                                             0.40202    0.03689  10.898
## ethnicityOther Hispanic                        -0.76180    1.98570  -0.384
## ethnicityNon-Hispanic White                    -2.50159    1.60537  -1.558
## ethnicityNon-Hispanic Black                     4.20990    1.66552   2.528
## ethnicityNon-Hispanic Asian                    -1.67884    1.89351  -0.887
## ethnicityOther Race - Including Multi-Racial    1.13619    3.06509   0.371
##                                               Pr(>|t|)    
## (Intercept)                                     <2e-16 ***
## age                                             <2e-16 ***
## ethnicityOther Hispanic                         0.7013    
## ethnicityNon-Hispanic White                     0.1194    
## ethnicityNon-Hispanic Black                     0.0116 *  
## ethnicityNon-Hispanic Asian                     0.3754    
## ethnicityOther Race - Including Multi-Racial    0.7109    
## ---
## Signif. codes:  0 '***' 0.001 '**' 0.01 '*' 0.05 '.' 0.1 ' ' 1
## 
## Residual standard error: 17.56 on 1602 degrees of freedom
## Multiple R-squared:  0.08829,    Adjusted R-squared:  0.08487 
## F-statistic: 25.85 on 6 and 1602 DF,  p-value: < 2.2e-16
```

```
## 
## Call:
## lm(formula = mean_sbp ~ age + ethnicity, data = subset(analysis_data, 
##     sex == "Female"))
## 
## Residuals:
##     Min      1Q  Median      3Q     Max 
## -55.674 -11.555  -1.790   9.955  88.881 
## 
## Coefficients:
##                                               Estimate Std. Error t value
## (Intercept)                                   85.46753    2.57855  33.146
## age                                            0.68901    0.03716  18.541
## ethnicityOther Hispanic                        0.23384    2.04744   0.114
## ethnicityNon-Hispanic White                   -0.07936    1.75086  -0.045
## ethnicityNon-Hispanic Black                    5.19739    1.77946   2.921
## ethnicityNon-Hispanic Asian                   -0.23415    2.01511  -0.116
## ethnicityOther Race - Including Multi-Racial  2.50774    3.56826   0.703
##                                               Pr(>|t|)    
```

```
## (Intercept)                                    < 2e-16 ***
## age                                            < 2e-16 ***
## ethnicityOther Hispanic                        0.90908
## ethnicityNon-Hispanic White                    0.96385
## ethnicityNon-Hispanic Black                    0.00354 **
## ethnicityNon-Hispanic Asian                    0.90751
## ethnicityOther Race - Including Multi-Racial  0.48229
## ---
## Signif. codes:  0 '***' 0.001 '**' 0.01 '*' 0.05 '.' 0.1 ' ' 1
##
## Residual standard error: 18.12 on 1661 degrees of freedom
## Multiple R-squared:  0.183,  Adjusted R-squared:  0.1801
## F-statistic: 62.02 on 6 and 1661 DF,  p-value: < 2.2e-16
```

```
# Males
# Model Coefficient Age
round(mod_summary_male$coefficients[2, ], 3)
```

```
##   Estimate Std. Error    t value   Pr(>|t|)
##      0.402      0.037     10.898      0.000
```

```
# 95% Confidence Interval Age
round(confint(regression_male), 3)[2,]
```

```
##  2.5 % 97.5 %
##  0.330  0.474
```

```
# Females
# Model Coefficient Age
round(mod_summary_female$coefficients[2, ], 3)
```

```
##   Estimate Std. Error    t value   Pr(>|t|)
##      0.689      0.037     18.541      0.000
```

```
# 95% Confidence Interval Age
round(confint(regression_female), 3)[2,]
```

```
##  2.5 % 97.5 %
##  0.616  0.762
```

**Males**: Per one year increase in age, systolic blood pressure will increase by 0.402 (95% CI: 0.330-0.474) units, given that all other covariates remain stable.

**Females**: Per one year increase in age, systolic blood pressure will increase by 0.689 (95% CI: 0.616-0.762) units, given that all other covariates remain stable.

These results suggest that systolic blood pressure increases more steeply with age in females than in males, indicating a potential sex difference in age-related blood pressure patterns.
