# Supplementary material for: Improving reproducibility of data analysis and code in medical research: 5 recommendations to get started
Source: BMJ Open. 2025 Oct 2;15(10):e104691. doi: 10.1136/bmjopen-2025-104691 (PMC12496075; doi:10.1136/bmjopen-2025-104691)
Supplement: Supplementary file 2 [file bmjopen-2025-104691.reviewer_comments.pdf]

PEER REVIEW HISTORY

BMJ Open publishes all reviews undertaken for accepted manuscripts. Reviewers are asked to complete a checklist review form and are provided with free text boxes to elaborate on their assessment. These free text comments are reproduced below.

ARTICLE DETAILS

Title (Provisional)

Improving Reproducibility of Data Analysis and Code in Medical Research - 5 Recommendations to Get Started

Authors

Streiber, Anna Maria; Hoepel, Sanne J.W.; Blok, Elisabet; van Rooij, Frank J.A; Neitzel, Julia; Labrecque, Jeremy; Ikram, M Kamram; Bos, Daniel

---

VERSION 1 - REVIEW

**Reviewer**	**1**
**Name**	**Dwivedi, Alok**
**Affiliation**	**Texas Tech University, Division of Biostatistics & Epidemiology, Department of Biomedical Sciences**
**Date**	**06-Jun-2025**
**COI**	**None**

Overall, the authors have provided a guidance document for preparing the statistical analysis codes and supplementing them with research publications. This is a nice attempt to improve the reproducibility of research data analysis in a publication. However, there are several aspects that need to be addressed in the manuscript to improve the purpose and reporting of statistical analysis code in a research publication.

1. The first recommendation is to make reproducibility and transparency a priority that also depends on how structured and comprehensively written data management and statistical analysis section in the manuscript and therefore, I recommend authors facilitate some discussion related to quality of writing statistical analysis section guidance documents such as “How to write statistical analysis section in medical research. Journal of Investigative medicine, 70(8), 1759-1770”, “Evidence-based statistical analysis and methods in biomedical research (SAMBR) checklists according to design features. Cancer Reports, 3(4), e1211.” And “the EQUATOR network and website. Evid Based Dent 8, 117 (2007)” so that readers focus on both the quality of reporting statistical analysis section to increase reliability and high reproducibility by facilitating statistical analysis codes.

2. A data dictionary with variable names is required, along with statistical codes to test and execute statistical codes properly.

3. The first section in Figure 1 should be code organization. I think the organization of the statistical code is required, such as data acquisition and cleaning, descriptive, unadjusted, final analyses, sensitivity/validation analyses, and exploratory analyses. The data example may be expanded to incorporate these additional analyses.

4. Within each code organization section, the label of the code should explain, such as Table 1, Table 2, Supplementary Tables 1 and 2, Figure 1, 2, or additional analysis (not reported in tables), etc., for a manuscript. So that readers may replicate specific tables/graphs and relate to the findings reported in a table or a graph.

5. The submission of the statistical code should not be restricted to one statistical package such as R. As long as the statistical codes are supplemented, that is sufficient, regardless of the statistical software.

6. In Figure 1 (comprehensibility), "Can the code be simplified?". This is not necessary as long as the code runs without an error and transparently provides results.

7. The check section could be written as a "clarity section" or "accountability" or "authenticity" section.

8. The comprehensibility section should include "Does the code incorporate all statistical methods reported in the statistical analysis section of the manuscript?". Although the authors have added an example under "Does the code run without error messages?", this should be checked if nothing in the code is missing that may yield an error in running the code.

9. The authors may want to recommend journal editors to make changes in journal policies to allow researchers to facilitate statistical codes along with a data dictionary. Furthermore, the authors may want to recommend which type of articles should incorporate the statistical analysis codes.

**Reviewer**	**2**
**Name**	**Cristall, Nora**
**Affiliation**	**University of Calgary Cumming School of Medicine, Medicine**
**Date**	**07-Jul-2025**
**COI**	**None**

This is an important topic in research. However, it is unclear what is meant by 'code' at the paper outset. The term should be clearly defined. Please build up to the conclusion to develop a checklist and not have this as the intent of the paper.

I would like to make the following recommendations:

1. The introduction in Lines 47 to 57 should be reordered and with more detail to make a more compelling argument at the outset to be concerned about coding reproducibility, The sentences at the end are better than the first two sentences.

2. The term ‘code’ should be articulated early in the paper. Are they also referring to how information is collected in a registry or repository? This is unclear to the reader.

3. The information under the heading Review of Coding Practices should include a more comprehensive examination of the topic in the literature.

4. The paper would be strengthened by a full disclosure of the authors orientation and training as many different types of researchers (qualitative coders, mixed methods coders etc.) are in the BMJ readership. What is their ontology and specific view in the research community?

5. Line 83 the word “to” should be removed.

6. In line 107 the authors should give examples of other disciplines.

7. The authors should introduce the checklist later in the paper and indicate how they concluded that this should be the best practice in coding.

8. On page 15 the authors should give the Checklist a clear title and define in the title what they are describing as code.

I hope that these comments are of assistance to the authors and BMJ in deciding whether to publish this paper.

Reviewer 3

Name Timm, Signe

Affiliation Lillebaelt Hospital - University Hospital of Southern Denmark, Oncology

Date 08-Jul-2025

COI **None**

The authors have made a clear, important and well-written paper with the vision of methodological rigor and enhanced reproducibility in statistical analyses. The recommendations are thorough and very time consuming, but I definitely agree with the title, that this is a way to get started.

I enjoyed reading it, and have some comments for further improvement and adjustment.

Introduction:

Have you made a literature review to search for similar guideline/recommendations on reproducibility? What does the literature say? If there are currently no guidance on this matte state this more directly.

Recommendation #1:

Line 93-94 says that code sharing is already well-established practice. That is not the impression from the introduction stating that code-sharing is only the case for 0.5% (line 53-54). Please clarify.

Recommendation #2:

What are unit tests (is this only applicable in R)? Please elaborate.

I agree that code review is ideal, however very time consuming. If a code reviwer is not available, you state that one can review own code. Please elaborate on the precautions to take in that case.

Code checklist is a very practical and concrete way to perform the code review. I like the idea of making it as easy for the code reviewer to perform the review. I have the following comments:
- include terms, that apply to other software than R (i.e. STATA) to make the checklist more generic (Check point 1 and 4, Transparency point 1). This applies for the main text too.
- include SAP in Check point 3 and Comprehensibility point 1
- include precautions about sharing microdata in Transparency point 5
- consider to mark the points 1.1, 1.2, 1.3 …

Recommendation #3:

I do not agree that coding efficiency is a goal in itself – as long as the analyses and coding are correct. Can you please elaborate on this point (Box 1, point 2).

Recommendation #4 & #5:

Ideally, the statistical analyses planed in the SAP should be numbered according to the research question (like question 1, 2, 3) and with sensitivity analyses, subgroup analyses ect. numbered (1.1, 1.2, 2.1 ..). Likewise, the coding file should be structured according to the numbered research questions, so that the code reviewer or the reader easily could follow which research questions is being answered down the coding file. Together with the availability of the code and data, the SAP should always be available too (published separately or not). Please include the connection between SAP and coding file.

Please include precautions on sharing microdata under #5.

Other comments:

The review of current coding practices is based solely on The Rotterdam Study. Are you aware of any other cohort studies with a long history of follow-up to compare with?

I miss a discussion section that critically discuss the treats and limitations of this recommendations being followed – i.e. funding issues, time issues ect. You have kind of it in the last sections, but call this "conclusion". In addition, how does these recommendations play together with the existing body of guideline papers i.e. for reporting. You touch upon STROBE, but I assume these recommendations applies to all kinds of study designs including trials, meta-analyses etc.

I agree that reproducibility should be a priority, however, do you have any comments or thoughts on how to secure funding for this part?

You state "analysis plan" several times, but I guess the right term is "statistical analyses plan" (SAP)

I am happy to read the revised version of this paper!

VERSION 1 - AUTHOR RESPONSE

Reviewer 1 (Dr. Alok Dwivedi):

Overall, the authors have provided a guidance document for preparing the statistical analysis codes and supplementing them with research publications. This is a nice attempt to improve the reproducibility of research data analysis in a publication. However, there are several aspects that need to be addressed in the manuscript to improve the purpose and reporting of statistical analysis code in a research publication.

1. The first recommendation is to make reproducibility and transparency a priority that also depends on how structured and comprehensively written data management and statistical analysis section in the manuscript and therefore, I recommend authors facilitate some discussion related to quality of writing statistical analysis section guidance documents such as "How to write statistical analysis section in medical research. Journal of Investigative medicine, 70(8), 1759-1770", "Evidence-based statistical analysis and methods in biomedical research (SAMBR) checklists according to design features. Cancer Reports, 3(4), e1211." And "the EQUATOR network and website. Evid Based Dent 8, 117 (2007)" so that readers focus on both the quality of reporting statistical analysis section to increase reliability and high reproducibility by facilitating statistical analysis codes.

Thank you for these suggestions. We fully agree that the reproducibility of a manuscript not only depends on the quality of the statistical code but on the statistical code in combination with a detailed explanation of the study's methodology. In line with the comments from Reviewer 2 and Reviewer 3, we have added a discussion section to the revised version of our manuscript. In that section, we elaborate on your suggestion as follows:

Lines 244-252: "***While reproducible code is a crucial part of reproducible medical research, it should be implemented in conjunction with reproducible documentation of research methods in an analysis plan and manuscript. More detailed recommendations for***

reproducible reporting of medical research are provided elsewhere, especially within the EQUATOR framework ((https://www.equator-network.org/) [3, 4]. Critical components in the documentation of statistical methods include detailed descriptions of variable selection processes, the statistical tests including assumption checks, potential sensitivity analyses, and the methods used for data presentation[30]. Detailed reporting of statistical methods may be further enhanced by using a corresponding checklist (e.g.[31]).*"*

2. A data dictionary with variable names is required, along with statistical codes to test and execute statistical codes properly.

We agree that adding a data dictionary is important to enhance comprehensibility and, thus, reproducibility of code. In Box 1, bullet point 1, we state that:

"*Comprehensibility can be improved by using consistent and clear names for datasets, variables, and other objects, and by providing a data dictionary. In a data dictionary, one describes the variables in the dataset in more detail*".

To further emphasize the importance of a data dictionary, we have added this to our code checklist (Figure 1) and incorporated it into our main text in line 160:

"*A first step is to use a clear structure, for example with adequate use of headings, a 'ReadMe' file explaining the workflow,* ***and a data dictionary describing the variable names***"

3. The first section in Figure 1 should be code organization. I think the organization of the statistical code is required, such as data acquisition and cleaning, descriptive, unadjusted, final analyses, sensitivity/validation analyses, and exploratory analyses. The data example may be expanded to incorporate these additional analyses.

In response to your feedback, as well as that of Reviewers 2 and 3, we have substantially restructured the code checklist. We agree that code organization is essential, particularly for comprehensibility, which is why we have added it as the first point under the "Comprehensibility" section. In that context, we also address one of your later comments regarding aligning the structure of the code with the structure of the manuscript.

We also appreciated your suggestion to incorporate this in our practical example and have revised the data example accordingly. We added a dedicated header for "Data Acquisition and Data Cleaning," introduced headers and examples for both sensitivity and exploratory analyses, and included specific examples: a sensitivity analysis excluding outliers, and an exploratory analysis stratifying by sex (Supplement 1).

4. Within each code organization section, the label of the code should explain, such as Table 1, Table 2, Supplementary Tables 1 and 2, Figure 1, 2, or additional analysis (not reported in tables), etc., for a manuscript. So that readers may replicate specific tables/graphs and relate to the findings reported in a table or a graph.

We agree that the code should refer to the sections of the paper as accurately as possible to facilitate reproducibility. We have now changed the headings in our code example accordingly (e.g.: “2.2.1 Model Building”, “2.2.2 Assumption check”, “2.2.3 Output Table 2” instead of “Code”, “Assumption check”, and “Output”, respectively). We further added the following to the Methods section of our code example (Supplement 1):

“*The accompanying R script clearly specifies which elements are included in a hypothetical manuscript or analysis plan and which are not. It follows the structure of a manuscript and uses consistent code labels that correspond to the names of tables and figures. We recommend this practice, as it facilitates reproducibility and improves comprehensibility*”.

We kindly refer to the revised version of Supplement 1 for a complete overview of all changes.

5. The submission of the statistical code should not be restricted to one statistical package such as R. As long as the statistical codes are supplemented, that is sufficient, regardless of the statistical software.

We fully agree that our recommendations are broadly applicable and are not limited to R. To address your comment, we have made the following changes to our manuscript:

Lines 66-69: “**An illustrative example of several of these reproducible coding practices is available in Supplement 1. While this example is written in R - a common programming language in medical research [12] - the structure and recommendations generalize to other coding languages. ”**

Box 1 bullet point 3: “Examples are using comments to explain the purpose of a code section or making use of documentation tools, such as ***Markdown*** (see Supplement 1) **or *Jupyter.***”

Furthermore, we have removed all recommendations that were phrased in a manner specific to R to make our methods more broadly applicable.

6. In Figure 1 (comprehensibility), “Can the code be simplified?”. This is not necessary as long as the code runs without an error and transparently provides results.

We agree that simplification of the code should not be a goal in itself, as long as the code is comprehensible, transparent, and conceptually correct. However, we have seen examples in our archive, where the code is highly inefficient and thus becomes long (> 10 000 lines) and incomprehensible, making a sound code review virtually impossible. Therefore, we recommend simplifying code where possible if it improves the comprehensibility and reusability. We added this nuance to our recommendation:

Figure 1: “*Can the code be simplified to enhance comprehensibility*?”

7. The check section could be written as a “clarity section” or “accountability” or “authenticity” section.

The purpose of the "check" column was to give researchers a simple way to tick off items of the checklist they had applied. However, we realized this was not entirely clear. We have therefore restructured the checklist by removing the "check" column and instead added icons and corresponding tick boxes directly within each row, making the checklist more intuitive and easier to use (Figure 1).

8. The comprehensibility section should include "Does the code incorporate all statistical methods reported in the statistical analysis section of the manuscript?". Although the authors have added an example under "Does the code run without error messages?", this should be checked if nothing in the code is missing that may yield an error in running the code.

This is an important point and we agree that this should be emphasized more in our recommendations. In our view, this constitutes two separate recommendations: First, structuring the code following the structure of the manuscript is an important way to improve comprehensibility. Second, checking whether the code incorporates all statistical methods reported in the analysis plan or methods section of the manuscript is crucial to check for conceptual correctness (i.e. whether the code does what it is supposed to do).

We have made this more explicit in our checklist by rewriting two of the recommendations:

(Comprehensibility; #1): "*Does the code follow the structure of the manuscript or analysis plan? Tip: Use (numbered) headings that directly refer to sections, tables, and figures of the manuscript*"

(Conceptual correctness, #2): "*Does the code incorporate all statistical methods reported in the manuscript or analysis plan?"*

Moreover, we have rearranged the order of the recommendations under *Conceptual Correctness* so that the recommendation to check whether the code runs without error messages is now placed after the recommendation to check whether all essential components are present, to reflect the logic of the comment of the reviewer.

9. The authors may want to recommend journal editors to make changes in journal policies to allow researchers to facilitate statistical codes along with a data dictionary. Furthermore, the authors may want to recommend which type of articles should incorporate the statistical analysis codes.

We agree that outlining specific recommendations for publishers and other third parties will be more insightful. Therefore, we have adapted our discussion accordingly.

Lines 256-260: "***Publishers should facilitate and encourage the upload of code at the submission state or facilitate easy linkage to public repositories such as ZENODO. Funding agencies can support reproducibility by allocating funding specifically to the dissemination***

of code and data or the implementation of code review, acknowledging that this will increase research quality and output downstream."

We now address article types in the final paragraph of our adapted discussion.

Lines 262-267: "***The Recommendations in this work are based on our review of the archive of the Rotterdam Study. Although we are not aware of existing archives with a similar set-up and length of follow-up, future work could expand our recommendations by surveying coding practices in other medical research contexts, e.g. from clinical trials.*** *There is also a need for systematic evidence on the effectiveness of practices such as code review in improving research reproducibility and quality*."

Reviewer 2 (Dr. Nora Cristall):

Thank you for asking me to review this paper. I would like to make the following recommendations:

1. The introduction in Lines 47 to 57 should be reordered and with more detail to make a more compelling argument at the outset to be concerned about coding reproducibility, The sentences at the end are better than the first two sentences.

We appreciate this suggestion and have rewritten the first paragraph of our introduction to better frame the relevance and urgency of our article:

Lines 47-58: ***"The reproducibility of research findings is a key part of the scientific method. Until recently, the reproducibility of medical research – i.e. whether an independent analysis of the same data yields consistent findings [1, 2] - mainly depended on the detailed description of the methods in the research paper. However, with the growing use of high dimensional data, recent methodological advances, and increasing occurrence of multi-cohort or multi-site studies, medical research has become increasingly complex. In this context, reproducibility of research strongly depends on the reproducibility of the code used in research, i.e. scripts written in programming languages such as R or Python that are used to preprocess data, derive analytical datasets from larger repositories and to perform statistical analyses [1, 3, 4]. However, Hamilton and colleagues [5] estimated that less than 0.5% of medical research studies that were published since 2016 shared their analytical code. Furthermore, reviews estimate that of papers that share code and data only a fraction is reproducible, with estimates ranging widely between 17-82% [6-8].***

2. The term 'code' should be articulated early in the paper. Are they also referring to how information is collected in a registry or repository? This is unclear to the reader.

We have now articulated our definition of code in the introduction:

Lines 52-55 "*In this context, reproducibility of research strongly depends on the reproducibility of the code used in research*, ***i.e. scripts written in programming languages***

such as R or Python that are used to preprocess data, derive analytical datasets from larger repositories and to perform statistical analyses [1, 3, 4]"

3. The information under the heading Review of Coding Practices should include a more comprehensive examination of the topic in the literature.

While preparing this manuscript we performed a comprehensive review of this topic in the literature, focusing especially on reproducibility of coding practices within medical research. Several guidelines exist to guide authors towards more reproducible research practices, but these recommendations often overlook the importance of coding practices and rarely provide concrete, practice-oriented examples. This prompted us to perform a review of real-world coding practices and provide researchers with actionable steps to improve the reproducibility of their code.

We believe that an extensive review of the literature is beyond the scope of this short communications paper and would not benefit the accessibility for the target audience. Nevertheless, we have now added the following concise paragraph to the section in question to better contextualize our observations within the broader literature and emphasize that our work addresses a gap in the existing literature, by providing concrete, practice-oriented examples:

Lines 72-83: "***Reproducibility in epidemiologic research is closely tied to the quality and transparency of analytical code. However, several studies have highlighted persistent shortcomings in current practices. Petrone et al. showed how minor differences in operational study definitions, such as cohort selection procedures, can lead to substantially different results, underscoring the need for precise and transparent reporting of coding decisions [13]. Similarly, Laurinavichyute and colleagues emphasized that sharing well-documented code is crucial for reproducibility and identified common barriers, including outdated code, missing preprocessing scripts, and insufficient documentation [7]. A recent review of over 9000 R scripts from replication datasets found that the majority failed to run in clean environments, illustrating widespread technical and documentation issues [14]. While these studies demonstrate that reproducibility can be improved through better coding practices, they do not always provide an in-depth evaluation of qualitative aspects of the code. To address this, we reviewed the internal code archive of the Rotterdam Study***"

Moreover, we refer to relevant previous publications throughout this manuscript (e.g. lines 134-137): "*Code review benefits reproducibility, as it ensures adherence to coding standards and improves code quality [17]. The code author will be more thorough when they know the code will be reviewed and the code reviewer can suggest improvements for transparency and comprehensibility of the code [20].*").

Last, in response to this comment and others, we now explicitly refer the reader to guidelines that focus on other aspects of reproducibility, such as reporting within a manuscript:

Lines 246-250: "***More detailed recommendations for reproducible reporting of medical research are provided elsewhere, especially within the EQUATOR framework ((https://www.equator-network.org/) [3, 4]. Critical components in the documentation of statistical methods include detailed descriptions of variable selection processes, the statistical tests including assumption checks, potential sensitivity analyses, and the methods used for data presentation [30]. Detailed reporting of statistical methods may be further enhanced by using a corresponding checklist (e.g. [31]).***"

4. The paper would be strengthened by a full disclosure of the authors orientation and training as many different types of researchers (qualitative coders, mixed methods coders etc.) are in the BMJ readership. What is their ontology and specific view in the research community?

We agree that providing additional information on the researchers' background helps the readers of this journal to better understand the perspective and motivation behind this article. To address this, we adapted the section on the review of coding practices as follows:

Lines 86- 90: "*For each Rotterdam Study publication, a folder is stored with the original data files and scripts or syntax files necessary for performing the statistical analyses.* ***As a team comprised of junior and senior epidemiologists and a data management specialist, we surveyed this archive to better understand how analytical code is written and maintained in real-world epidemiologic research***."

Additionally, we now articulate in the discussion that further work is needed to make our recommendations generalizable to the diverse range of medical research that is performed in the field:

Lines 262-267: "***The recommendations in this work are based on our review of the archive of the Rotterdam Study. Although we are not aware of existing archives with a similar set-up and length of follow-up, future work could expand our recommendations by surveying coding practices in other medical research contexts, e.g. from clinical trials.*** *There is also a need for systematic evidence on the effectiveness of practices such as code review in improving research reproducibility and quality.*"

5. Line 83 the word "to" should be removed.

Thank you for pointing this out, we have adapted this line accordingly.

6. In line 107 the authors should give examples of other disciplines.

We have adapted the corresponding sentence as follows:

Lines 126-127: "*Preliminary findings from other disciplines, **such as evolutionary biology**, suggest that papers with shared code and data may accumulate citations faster [19]*"

7. The authors should introduce the checklist later in the paper and indicate how they concluded that this should be the best practice in coding.

We agree that the checklist follows from our code review and listed recommendation and should, thus, be presented as a conclusion. We have restructured our paper accordingly and now separately discuss the code checklist after recommendation 5. We have removed any references to the code checklist from earlier sections. We have also extended our description of the checklist slightly to improve interpretability:

Lines 223-235: ***"Based on the recommendations outlined before, we developed a code checklist (Figure 1) aimed at facilitating the implementation of code review. This checklist can be used during code review and serves two separate aims, which are reflected in its two parts. First, the checklist is used to assess the reproducibility of the code, focusing on our recommendations for writing comprehensible and transparent code. Second, the checklist provides guiding questions that can be used to assess the conceptual correctness of the code (i.e. does the code do what it is supposed to do?). Here, the code reviewer focuses on whether the code performs the analyses that are outlined in the accompanying statistical analysis plan or paper in the correct way. The checklist contains practical tips to improve code and leaves room for comments that arise during the review process."***

8. On page 15 the authors should give the Checklist a clear title and define in the title what they are describing as code.

Based on our refined definition of "code", have now changed the title of the code checklist to "***Code review checklist to assess reproducibility and conceptual correctness of code used for data preprocessing, dataset generation, and statistical analyses***".

Reviewer 3 (Signe Timm):

The authors have made a clear, important and well-written paper with the vision of methodological rigor and enhanced reproducibility in statistical analyses. The recommendations are thorough and very time consuming, but I definitely agree with the title, that this is a way to get started.

I enjoyed reading it, and have some comments for further improvement and adjustment.

Introduction:

Have you made a literature review to search for similar guideline/recommendations on reproducibility? What does the literature say? If there are currently no guidance on this matte state this more directly.

While preparing this manuscript we performed a comprehensive review of this topic in the literature, focusing especially on reproducibility of coding practices within medical research. Several guidelines exist to guide authors towards more reproducible research practices, but these recommendations often overlook the importance of coding practices and rarely provide concrete, practice-oriented examples. This prompted us to perform a review of real-world coding practices and provide researchers with actionable steps to improve the reproducibility of their code.

We believe that an extensive review of the literature is beyond the scope of this short communications paper and would not benefit the accessibility for the target audience. Nevertheless, we have now added the following concise paragraph to the section in question to better contextualize our observations within the broader literature and emphasize that our work addresses a gap in the existing literature, by providing concrete, practice-oriented examples:

Lines 72-83: "***Reproducibility in epidemiologic research is closely tied to the quality and transparency of analytical code. However, several studies have highlighted persistent shortcomings in current practices. Petrone et al. showed how minor differences in operational study definitions, such as cohort selection procedures, can lead to substantially different results, underscoring the need for precise and transparent reporting of coding decisions [13]. Similarly, Laurinavichyute and colleagues emphasized that sharing well-documented code is crucial for reproducibility and identified common barriers, including outdated code, missing preprocessing scripts, and insufficient documentation [7]. A recent review of over 9000 R scripts from replication datasets found that the majority failed to run in clean environments, illustrating widespread technical and documentation issues [14]. While these studies demonstrate that reproducibility can be improved through better coding practices, they do not always provide an in-depth evaluation of qualitative aspects of the code. To address this, we reviewed the internal code archive of the Rotterdam Study."***

Moreover, we refer to relevant previous publications throughout this manuscript (e.g. lines 134-137): "*Code review benefits reproducibility, as it ensures adherence to coding standards and improves code quality [17]. The code author will be more thorough when they know the code will be reviewed and the code reviewer can suggest improvements for transparency and comprehensibility of the code [20]*.").

Last, in response to this comment and others, we now explicitly refer the reader to guidelines that focus on other aspects of reproducibility, such as reporting within a manuscript:

Lines 246-252: "***More detailed recommendations for reproducible reporting of medical research are provided elsewhere, especially within the EQUATOR framework ((https://www.equator-network.org/) [3, 4]. Critical components in the documentation of***

statistical methods include detailed descriptions of variable selection processes, the statistical tests including assumption checks, potential sensitivity analyses, and the methods used for data presentation [30]. Detailed reporting of statistical methods may be further enhanced by using a corresponding checklist (e.g. [31]).”

Recommendation #1:

Line 93-94 says that code sharing is already well-established practice. That is not the impression from the introduction stating that code-sharing is only the case for 0.5% (line 53-54). Please clarify.

We agree that our phrasing in Recommendation 1, may seem contradicting to our introduction. We have now changed the phrasing as follows:

Lines 111-113: “*For this reason, reproducible coding practices, especially sharing data and code, are **frequently recommended** open science practices*.”

Recommendation #2:

What are unit tests (is this only applicable in R)? Please elaborate.

In statistical programming, unit tests are small tests that check whether a specific function or code chunk works as intended under defined conditions. They can be implemented in any programming language and are a general tool for validating code functionality. For instance, in Supplement 1, we integrated a unit test to check if our function to compute the mean blood pressure works as intended. As this is a technical term, we decided to further explain it in our manuscript:

Lines 148-149: “***Unit tests are automated checks to verify that individual parts of the code, such as functions or processing steps, perform as intended***.”

I agree that code review is ideal, however very time consuming. If a code reviewer is not available, you state that one can review own code. Please elaborate on the precautions to take in that case.

We have extended our discussion of the code checklist and added a discussion of the potential pitfalls of using the checklist for self-review:

Lines 233-235: “*Additionally, one can use the code checklist to perform a structured self-review, but this approach is more likely to overlook reproducibility issues, due to the reviewer’s familiarity with the code’s logic and environment*.”

Code checklist is a very practical and concrete way to perform the code review. I like the idea of making it as easy for the code reviewer to perform the review. I have the following comments:

- include terms, that apply to other software than R (i.e. STATA) to make the checklist more generic (Check point 1 and 4, Transparency point 1). This applies for the main text too.

We have carefully adjusted our manuscript and example to emphasize the broad applicability of our recommendations, beyond R:

Lines 66-69: "***An illustrative example of several of these reproducible coding practices is available in Supplement 1. While this example is written in R - a common programming language in medical research - the structure and recommendations generalize to other coding languages.*** "

Box 1 bullet point 3: "*Examples are using comments to explain the purpose of a code section or making use of documentation tools, such as* ***Markdown*** *(see Supplement 1)* ***or Jupyter.***"

Furthermore, we have removed all recommendations that were phrased in a manner specific to R to make our methods more broadly applicable. We have decided against further including terms that are specific to other software (i.e. STATA or Python), to keep the text easily accessible for a wide range of researchers.

- include SAP in Check point 3 and Comprehensibility point 1

We have updated our checklist (Figure 1) and now recommend that the code follows the structure of the manuscript or analysis plan (Comprehensibility, #1) and that the research aim or analysis plan is included in the annotated script so that the conceptual correctness of the code can be accessed (Conceptual Correctness, #1).

- include precautions about sharing microdata in Transparency point 5

We have added precautions about sharing microdata in our main text, as noted in more detail below.

We have additionally rewritten the recommendation under Transparency point 5 (sample selection), to avoid the impression that it is desirable to note any individual-level characteristics of in- or excluded participants:

Figure 1: "*Tip: Note step by step which* ***groups of*** *participants are excluded and why.*"

- consider to mark the points 1.1, 1.2, 1.3 …

We have restructured our code checklist to improve clarity, by changing the heading and replacing the 'check' column with an icon to indicate that researchers can check the box, when the suggestions from the checklist are implemented in the code. However, we deliberately decided against including numbering in the checklist, as this might give the impression that items are numbered in order of importance.

Recommendation #3:

I do not agree that coding efficiency is a goal in itself – as long as the analyses and coding are correct. Can you please elaborate on this point (Box 1, point 2).

We agree that simplification of the code should not be a goal in itself, as long as the code is comprehensible, transparent, and conceptually correct. However, we have observed examples in our archive, where the code is highly inefficient and thus becomes long (> 10 000 lines) and incomprehensible, making a sound code review virtually impossible. Therefore we recommend simplifying code where possible if it improves the comprehensibility. We added this nuance to our recommendation:

Figure 1, Comprehensibility #3: "*Can the code be simplified* ***to enhance comprehensibility?***"

Recommendation #4 & #5: Ideally, the statistical analyses planed in the SAP should be numbered according to the research question (like question 1, 2, 3) and with sensitivity analyses, subgroup analyses ect. numbered (1.1, 1.2, 2.1 ..). Likewise, the coding file should be structured according to the numbered research questions, so that the code reviewer or the reader easily could follow which research questions is being answered down the coding file. Together with the availability of the code and data, the SAP should always be available too (published separately or not). Please include the connection between SAP and coding file.

We agree that the coding file should be structured as similar to the analysis plan and manuscript as possible. However, we do not want to specifically recommend doing this with numbering, as this approach might not be applicable to all types of medical research. To illustrate this in our example, we have added the following to the Methods section of our code example:

Supplement 1: "***The accompanying R script clearly specifies which elements are included in a hypothetical manuscript or analysis plan and which are not. It follows the structure of a manuscript or analysis plan and uses consistent code labels that correspond to the names of tables and figures. We recommend this practice, as it facilitates reproducibility and improves comprehensibility***".

In addition, we added a dedicated header for "Data Acquisition and Data Cleaning," introduced headers and examples for both sensitivity and exploratory analyses, and included specific examples: a sensitivity analysis excluding outliers, and an exploratory analysis stratifying by sex (Supplement 1).

Furthermore, we now underscore the importance of reproducible reporting in an analysis plan and in the manuscript in our discussion:

Lines 244-246: "***While reproducible code is a crucial part of reproducible medical research, it should be implemented in conjunction with reproducible documentation of research methods in an analysis plan and manuscript***."

Please include precautions on sharing microdata under #5.

We agree that data sharing should always be in line with privacy regulations and have now added this to our recommendations:

Lines 193-197: "*It may not always be possible to share analytical data and* ***when individual-level participant data is shared, researchers should be mindful that this in compliance with participant consent and local data protection laws. They should take precautions to protect privacy and prevent re-identification, for example by masking quasi-identifiers and applying k-anonymity****.*"

Other comments:

The review of current coding practices is based solely on The Rotterdam Study. Are you aware of any other cohort studies with a long history of follow-up to compare with?

The Generation R cohort, which is also affiliated with the Erasmus Medical Center in Rotterdam, has adopted our archiving methods, but their follow-up is not as extensive as the Rotterdam Study. Surveying coding practices at other institutes or beyond cohort studies could improve the generalizability of our recommendations. We have expanded on this in our discussion:

Lines 262-265: **"*The recommendations in this work are based on our review of the archive of the Rotterdam Study. Although we are not aware of existing archives with a similar set-up and length of follow-up, future work could expand our recommendations by surveying coding practices in other medical research contexts, e.g. from clinical trials*."**

I miss a discussion section that critically discuss the treats and limitations of this recommendations being followed – i.e. funding issues, time issues ect. You have kind of it in the last sections, but call this "conclusion". In addition, how does these recommendations play together with the existing body of guideline papers i.e. for reporting. You touch upon STROBE, but I assume these recommendations applies to all kinds of study designs including trials, meta-analyses etc.

We agree that a more in-depth discussion of the strengths and limits of our recommendations is warranted. We have removed the summary of recommendations that constituted our conclusions and instead now discuss several points. We now note that while our recommendations focus on code, these should be implemented in conjunction with existing guidelines on statistical analysis plans and reporting:

Lines 244-252: "***While reproducible code is a crucial part of reproducible medical research, it should be implemented in conjunction with reproducible documentation of research methods in an analysis plan and manuscript. More detailed recommendations for reproducible reporting of medical research are provided elsewhere, especially within the EQUATOR framework ((https://www.equator-network.org/) [3, 4]. Critical components in the documentation of statistical methods include detailed descriptions of variable selection processes, the statistical tests including assumption checks, potential sensitivity***

analyses, and the methods used for data presentation [30]. Detailed reporting of statistical methods may be further enhanced by using a corresponding checklist (e.g. [31]).”

I agree that reproducibility should be a priority, however, do you have any comments or thoughts on how to secure funding for this part?

Throughout this paper our goal has been to provide researchers with actionable steps that can be implemented even in a context of limited resources. We firmly believe that writing reproducible code will, in fact, increase efficiency in research and is, thus, worth the time investment, even if not specifically funded. We do agree that funders can play a role by supporting this time investment. We have now added this to our discussion:

Lines 258-261: “***Funding agencies can support reproducibility by allocating dedicated resources to the dissemination of code and data or the implementation of code review, acknowledging that this will increase research quality and output downstream. For example, funding calls could explicitly include budget lines for reproducibility infrastructure.***”

You state “analysis plan” several times, but I guess the right term is “statistical analyses plan” (SAP)

We use the term analysis plan in accordance with terminology widely adopted in the reproducibility literature (e.g., https://doi.org/10.1186/s40359-016-0126-3; https://doi.org/10.1098/rsta.2020.0210), particularly in the context of preregistration and transparent reporting in observational research. The use of this term is also reflected in platforms such as the Open Science Framework, which consistently refers to “analysis plans” rather than “statistical analysis plans” (see: https://osf.io/zab38/wiki/home/). While Statistical Analysis Plan is a more formal and technical term typically used in clinical trials, our focus is broader and includes both statistical and procedural elements.

I am happy to read the revised version of this paper

VERSION 2 - REVIEW

**Reviewer**	**1**
**Name**	**Dwivedi, Alok**
**Affiliation**	**Texas Tech University, Division of Biostatistics & Epidemiology, Department of Biomedical Sciences**
**Date**	**02-Sep-2025**
**COI**	

The authors have incorporated all my suggestions in the revised manuscript and addressed them thoroughly. I have no further comments.

Reviewer **3**

Name **Timm, Signe**

Affiliation **Lillebaelt Hospital - University Hospital of Southern Denmark, Oncology**

Date **27-Aug-2025**

COI

Thank you for your response. I have no further comments to add.
